# Comparative Genomics of *Saccharomyces cerevisiae* Natural Isolates for Bioenergy Production

**DOI:** 10.1093/gbe/evu199

**Published:** 2014-09-05

**Authors:** Dana J. Wohlbach, Nikolay Rovinskiy, Jeffrey A. Lewis, Maria Sardi, Wendy S. Schackwitz, Joel A. Martin, Shweta Deshpande, Christopher G. Daum, Anna Lipzen, Trey K. Sato, Audrey P. Gasch

**Affiliations:** ^1^Laboratory of Genetics, University of Wisconsin, Madison; ^2^DOE Great Lakes Bioenergy Research Center, University of Wisconsin, Madison; ^3^US Department of Energy Joint Genome Institute, Walnut Creek, California; ^4^Present address: Biology Department, Dickinson College, Carlisle, PA; ^5^Present address: Department of Biological Sciences, University of Arkansas, Fayetteville, AR

**Keywords:** bioenergy, genomics, transcriptomics, environmental stress

## Abstract

Lignocellulosic plant material is a viable source of biomass to produce alternative energy including ethanol and other biofuels. However, several factors—including toxic byproducts from biomass pretreatment and poor fermentation of xylose and other pentose sugars—currently limit the efficiency of microbial biofuel production. To begin to understand the genetic basis of desirable traits, we characterized three strains of *Saccharomyces cerevisiae* with robust growth in a pretreated lignocellulosic hydrolysate or tolerance to stress conditions relevant to industrial biofuel production, through genome and transcriptome sequencing analysis. All stress resistant strains were highly mosaic, suggesting that genetic admixture may contribute to novel allele combinations underlying these phenotypes. Strain-specific gene sets not found in the lab strain were functionally linked to the tolerances of particular strains. Furthermore, genes with signatures of evolutionary selection were enriched for functional categories important for stress resistance and included stress-responsive signaling factors. Comparison of the strains’ transcriptomic responses to heat and ethanol treatment—two stresses relevant to industrial bioethanol production—pointed to physiological processes that were related to particular stress resistance profiles. Many of the genotype-by-environment expression responses occurred at targets of transcription factors with signatures of positive selection, suggesting that these strains have undergone positive selection for stress tolerance. Our results generate new insights into potential mechanisms of tolerance to stresses relevant to biofuel production, including ethanol and heat, present a backdrop for further engineering, and provide glimpses into the natural variation of stress tolerance in wild yeast strains.

## Background

Lignocellulosic plant material represents an untapped feedstock for microbial biofuel production. However, extracting monomeric sugars in hemicellulose from the lignin and solid fraction often requires harsh chemical pretreatments that generate toxins inhibitory to microbial fermentation. The precise toxins generated vary by the pretreatment method as well as the plant source material, and emerge both from the treatment additives (such as strong acids, bases, or other chemicals) as well as chemical reactions with plant materials, including sugar-derived phenolic compounds (Almeida et al. 2007; Lau et al. 2009; Chundawat et al. 2010). The effect of these lignotoxins is compounded by high osmolarity of the resulting hydrolysates, elevated temperature of many fermentation processes, and ethanol generated during anaerobic fermentation (Jin et al. 2013; Sato et al. 2014). As such, the combined stresses in cellulosic fermentations represent a major bottleneck to efficient microbial conversion of biomass.

One strategy is to improve microbial stress tolerance via artificial, laboratory strain evolution, through many generations of selective growth in stressful conditions (Dragosits and Mattanovich 2013). Although improved tolerance to particular stresses can be selected in the lab, it often comes at the cost of reduced fitness in unstressed conditions and/or diminished biofuel production (Bennett and Lenski 2007; Goodarzi et al. 2010; Watanabe et al. 2011; Dragosits and Mattanovich 2013; Hong and Nielsen 2013). Furthermore, the limited mutational landscape that is accessible from a single starting strain prevents the broad sampling of genetic possibilities.

An alternative approach is to start with natural isolates that are inherently tolerant to relevant stresses, both to understand the mechanism of their stress tolerance and to exploit for further directed engineering. The genetic and phenotypic variation of *Saccharomyces cerevisiae* is beginning to emerge through studies of both wild and industrial yeast isolates (Townsend 2003; Aa et al. 2006; Kvitek et al. 2008; Liti et al. 2009; Borneman et al. 2011; Magwene et al. 2011; Warringer et al. 2011). *Saccharomyces cerevisiae* populations represent at least 13 distinct lineages, with many strains representing “mosaic” genomes due to recent, but likely infrequent, admixture across the well-separated lineages (Wei et al. 2007; Liti et al. 2009; Schacherer et al. 2009; Wang et al. 2012; Cromie et al. 2013). A vast amount of phenotypic diversity exists across these strains and in some cases correlates with the niche from which the strains were isolated (Kvitek et al. 2008; Will et al. 2010; Warringer et al. 2011). Understanding the genetic basis for natural variation in stress tolerance is in its infancy but is being aided by quantitative mapping within and between populations (reviewed in [Liti and Louis 2012]). However, the genetic basis for extreme tolerance remains poorly understood.

To address this question, we sequenced the genomes and transcriptomes of three natural *S. cerevisiae* isolates with extreme tolerance to stresses relevant to biofuel production, including two strains with high thermotolerance or high ethanol resistance and one multistress tolerant strain that was particularly amenable to growth in plant-derived hydrolysate. We report the genomic analysis of these isolates and implicate key physiological processes related to biofuel-relevant stress tolerance.

## Materials and Methods

### Yeast Strains

**Fig. 1.—:**
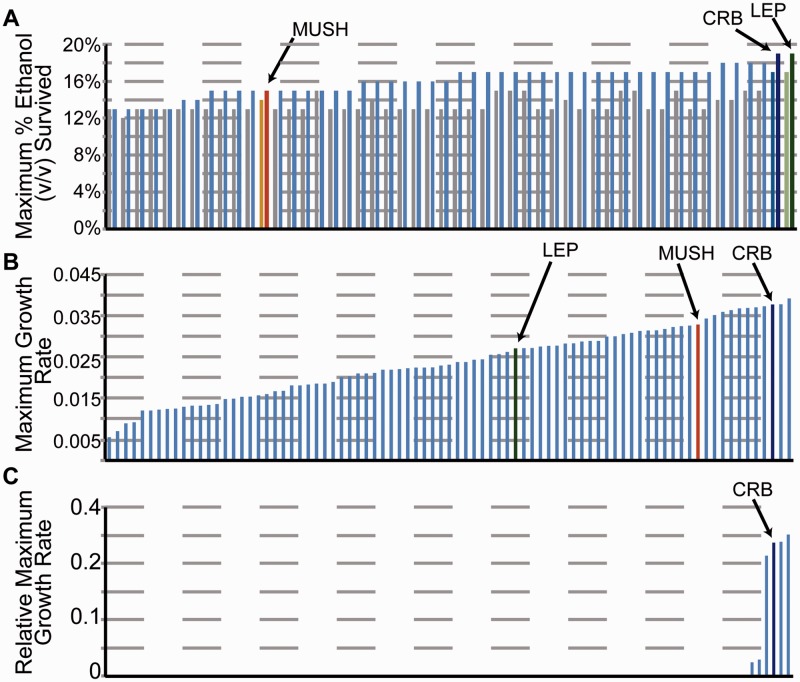
Stress tolerance profiles. (*A*) Acquired ethanol tolerance of LEP, MUSH, CRB, and other strains from (Lewis et al. 2010). Cells were exposed to high does of ethanol with (blue) or without (gray) prior treatment of 5% v/v ethanol. (*B* and *C*) Growth rates (see Materials and Methods) of *S. cerevisiae* strains in YPD at 40 °C (*B*) or in ACSH at 42 °C (*C*) relative to YPD (adapted from [Sato et al. 2014]).

Yeast strains were grown in yeast extract peptone dextrose (YPD; 10 g/l yeast extract, 20 g/l peptone, 20 g/l glucose) at 30 °C. For acquired ethanol resistance, cells were pretreated with 5% v/v for 60 min and then exposed to one of 11 doses of ethanol ranging from 5 to 25% v/v for 2 h before plating for viability (Lewis et al. 2010). The maximum dose of ethanol survived is plotted in figure 1. Growth rates under the other conditions were calculated based on 96-well growth profiles in a Tecan plate reader, using GCAT as previously described (Jin et al. 2013; Sato et al. 2014). Strain phenotypes are available in supplementary data set S4, Supplementary Material online.

**Fig. 2.—:**
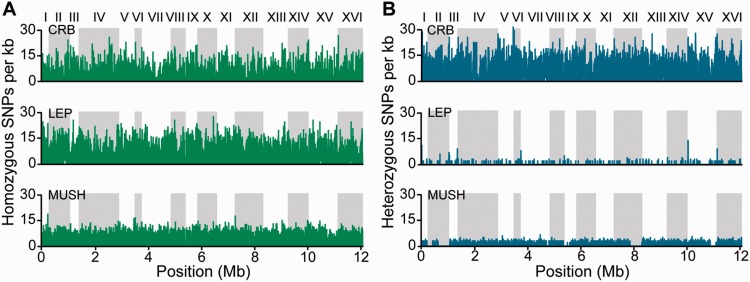
Distribution of SNPs across the genome. Homozygous (*A*, green) and heterozygous (*B*, blue) SNPs relative to the S288c reference for 16 *S. cerevisiae* chromosomes, with a sliding 1-kb window of 100 bp step size.

### Genome Sequencing, Read Mapping, and Single Nucleotide Polymorphism Calling

Libraries were generated with a modified version of Illumina’s standard protocol, using 1 µg of genomic DNA. DNA was sonicated (Covaris) to generate fragments, which were size selected by SPRI to approximately 200 bp. Selected fragments were end-repaired and phosphorylated, A-tailed with klenow, and ligated with paired-end sequencing adapters (Illumina). Libraries were PCR (polymerase chain reaction) amplified and quantified using KAPA Biosystem’s next-generation sequencing library qPCR kit and run on a Roche LightCycler 480 real-time PCR instrument. Each of the quantified sample libraries were prepared for sequencing utilizing a paired-end cluster generation kit (v4), and Illumina’s cBot instrument to generate clusters. Sequencing was performed on the Illumina GAIIx sequencer using SBS sequencing kits, v4, following a 2 × 76 run recipe.

Mapping was performed with Burrows–Wheeler Alignment (v1.2.2; [Li and Durbin 2009]), combining strains with multiple libraries (supplementary table S1, Supplementary Material online). Single nucleotide polymorphism (SNP) and indel detection was performed with the Genome Analysis Toolkit (v2.7; [McKenna et al. 2010]). De novo genome assembly was performed using String Graph Assembler (v0.9.19; [Simpson and Durbin 2012]), and contigs were aligned with MUMmer (v3.0; [Kurtz et al. 2004]; supplementary table S2, Supplementary Material online). Unique sequence not present in S288c was identified using custom Perl scripts. Gene prediction on non-S288c sequence was performed using GlimmerHMM (v3.0.2; [Majoros et al. 2004]) trained on *S. cerevisiae* transcripts. Additional details are available in supplementary methods, Supplementary Material online. Genome sequencing data for each strain are available (http://jgi.doe.gov/, last accessed September 15, 2014).

### Comparative Genomic Hybridization

Array-based comparative genomic hybridization (aCGH) was performed in biological duplicate on CRB, LEP, and MUSH relative to a DBY8268 control as previously described (Pollack et al. 1999). Samples were labeled using amino-allyl dUTP (Ambion), Klenow exo-polymerase (New England Biolabs), and random hexamers, and coupled with cyanine dyes (Amersham). Samples were hybridized to custom 385K tiling arrays (NimbleGen) designed using chipD (Dufour et al. 2010) on the composite *S. cerevisiae* genome described above. Arrays were hybridized in a NimbleGen hybridization system 12 (BioMicro) and scanned using a scanning laser (GenePix 4000B, Molecular Devices) according to NimbleGen protocols (http://www.nimblegen.com/, last accessed September 15, 2014). Data normalization was performed using Bioconductor (Gentleman et al. 2004) and custom Perl scripts. The affy() package (Gautier et al. 2004) was used to apply probe-level quantile normalization to the log_2_ ratios. We defined genes with increased copy number as those with a log_2_ aCGH ratio greater than 0.7 (because relative intensity values are often slightly compressed from the expected duplication log_2_ value of 1.0); genes with a log_2_ aCGH ratio < −1.0 were identified as potentially deleted. All microarray data are available through the NCBI Gene Expression Omnibus under the accession GSE56441.

### RNA-Seq Library Construction and Sequencing

Each strain was subjected to 25–37 °C heat shock for 15 min or 5% (v/v) ethanol treatment for 30 min. Cells were grown in YPD medium to log phase (OD_600_ ∼0.5) for at least four doublings, at which point a sample of unstressed cells was removed. RNA was extracted by hot phenol lysis (Gasch 2002) and mRNA was twice purified from total RNA using the Absolutely mRNA purification kit (Stratagene). mRNA samples were chemically fragmented to 200–250 bp using 1× fragmentation solution for 5 min at 70 °C (Ambion). First strand cDNA was synthesized using Superscript II Reverse Transcriptase (Invitrogen) and random hexamers. cDNA was purified with Ampure SPRI beads. The second strand was synthesized using dNTPs (dTTP replaced with dUTP), *Escherichia coli* RNaseH, DNA Ligase, and DNA polymerase I for nick translation. dscDNA were purified and selected for 200–300 bp fragments using a double Ampure SPRI bead selection, blunt-ended, poly A tailed, and ligated with Truseq adaptors using the Illumina DNA Sample Prep Kit (Illumina). Adaptor-ligated DNA was purified using Ampure SPRI beads. The second strand was removed by AmpErase UNG (Applied Biosystems) using a method similar to (Parkhomchuk et al. 2009). Digested cDNA was cleaned with Ampure SPRI beads. Paired-end 76 bp reads were generated by sequencing using the Illumina HiSeq instrument.

### RNA-Seq Read Processing, De Novo Assembly, and Counting

Reads were filtered and mapped as described above. Differential expression analysis was performed with edgeR() (Robinson et al. 2010), using a general linearized model comparing expression in each strain relative to the average expression pattern across strains, with strain background and environmental condition as factors and pairing replicate samples. De novo transcript identification was performed using Trinity (Grabherr et al. 2011). Resulting transcripts are available in supplementary data set S5, Supplementary Material online.

For other expression analysis, genes with fewer than ten mapped reads in at least one experiment were removed from subsequent analysis. These data were subjected to model-based clustering using mclust() (Fraley et al. 2012) with the VII model and *k* = 100. Expression data were visualized using Java TreeView (Saldanha 2004). Enriched gene ontology (GO) categories were assigned using FunSpec or GO-TermFinder (Robinson et al. 2002; Boyle et al. 2004). Additional details are available in supplementary methods, Supplementary Material online. Processed expression data are available in supplementary data sets S6 and S7, Supplementary Material online, respectively. Transcript sequencing data are available through the NCBI Sequence Read Archive under the accessions SRA051794 and SRA146858 (S288c), SRA051792 and SRA146754 (CRB), SRA051801 and SRA146751 (LEP), and SRA149355 (MUSH).

### Population Genomics Analysis

We obtained whole-genome sequence from 63 additional strains of *S. cerevisiae* (supplementary table S3, Supplementary Material online) and performed pairwise whole-genome alignments relative to the reference strain S288c with MUMmer (v3.0; [Kurtz et al. 2004]). We constructed a neighbor-joining tree from all SNPs using PHYLIP (v3.6; [Felsenstein 1989]) and used Structure (v2.3.2.1; [Pritchard et al. 2000]) to infer the population history with 11,795 SNPs distributed approximately evenly across the genome (∼1 SNP/1 kb). We tested *K* from 1 to 7 under the linkage model for 50,000 iterations, after a burn-in of 20,000 to 40,000 iterations. We estimated the posterior probability for each *K* by assuming a uniform prior on *K* = {1, … , 7} and determined that *K = 6* is the most probable model that captures the majority of structure in the data. We calculated the nonsynonymous to synonymous substitution rate (*K*_a_/*K*_s_) using the KaKsCalculator (v2.0; [Wang et al. 2010]) under the MA model.

## Results

Through the course of various phenotyping efforts, we identified three natural-isolate, diploid strains of *S. cerevisiae* that were tolerant to stresses relevant to biofuel production. Strains Y-2209 (“LEP,” isolated from *Lepidopterous* in California) and Y-389 (“MUSH,” isolated from mushrooms) were identified as being among the more tolerant to ethanol treatment and high temperature, respectively (fig. 1). Strain YB-210 (“CRB,” isolated from banana in Costa Rica) was tolerant to several stresses, including ethanol, heat, acetic acid, phenolics, and sodium, as well as ammonia fiber-expansion (AFEX)-treated corn stover hydrolysate (ACSH; [Jin et al. 2013] and fig. 1). CRB, and to some extent MUSH, were also among the strains that could grow well at 40 °C and in ACSH at 42 °C (fig. 1).

### Genome Sequencing Uncovers Extensive Genome Differences

To examine the genotypes of these strains, we sequenced their genomes with high-depth (>200×) short-read (76 bp) Illumina sequencing (see Materials and Methods). After mapping to a composite reference genome representing multiple strains, we identified approximately 44,000–78,000 SNPs and approximately 5,000–10,000 indels for each strain relative to the reference genome (table 1 and supplementary table S4, Supplementary Material online). Approximately 82% of these SNPs have been observed in least one other sequenced strain (Goffeau et al. 1996; Wei et al. 2007; Borneman et al. 2008, 2011; Doniger et al. 2008; Argueso et al. 2009; Novo et al. 2009; Dowell et al. 2010; Akao et al. 2011; Babrzadeh et al. 2012). Of the 21,601 novel SNPs, all but 603 are found in only one of our strains, suggesting they are either false-positive identifications, have emerged through new mutation, or represent polymorphisms present in populations for which complete genome sequence is not yet available (e.g., recent Chinese isolates; [Wang et al. 2012]; supplementary fig. S1, Supplementary Material online). Notably, MUSH contains the largest number of novel SNPs (supplementary table S5, Supplementary Material online).

**Table 1:** Genomic Distribution of Intergenic (I), Synonymous (S), and Nonsynonymous (NS) SNPs

	CRB			LEP			MUSH		
	Hom.	Het.	Total	Hom.	Het.	Total	Hom.	Het.	Total
I	8,849	15,806	24,655	15,439	119	15,437	9,328	17,150	26,478
S	10,432	20,740	31,172	18,192	59	18,251	9,900	22,281	32,181
NS	6,102	10,592	16,694	10,518	89	10,607	6,310	13,085	19,395
Tot.	25,383	47,138	72,521	44,149	267	44,416	25,538	52,516	78,054

Note.—Hom., homozygous; Het., heterozygous.

Heterozygous sites were found in all three diploid sequenced strains; however, there was a wide distribution in the level of heterozygosity, spanning the range measured in other sequenced strains (supplementary table S6, Supplementary Material online; [Akao et al. 2011; Borneman et al. 2011; Magwene et al. 2011; Babrzadeh et al. 2012]). LEP is almost entirely homozygous, with only 267 heterozygous sites (table 1). Although both MUSH and CRB are highly heterozygous, (67% and 65% biallelic SNPs, respectively; table 1), MUSH also contains several regions of loss of heterozygosity (LOH; fig. 2 and supplementary note S1, Supplementary Material online). The genome-wide heterozygosity of CRB may explain the low spore viability of this diploid strain (∼2% viability), which could mask recessive lethal alleles that are uncovered in spores.

We also performed aCGH to assess copy number variation (CNV) across individual genes (see Materials and Methods) and observed extensive CNV in CRB, LEP, and MUSH relative to the diploid reference lab strain DBY8628, most notably in subtelomeric regions, which are known to be enriched for genes involved in stress response and carbohydrate metabolism ([Brown et al. 2010]; see supplementary note S2 and fig. S2, Supplementary Material online).

### Nonrandom SNP Distributions Suggest Signatures of Selection

For all three strains, approximately one-third of detected SNPs were intergenic (table 1), whereas only about 25% of the *S. cerevisiae* genome is noncoding (Cherry et al. 2012). This indicates a significant skew toward intergenic SNPs in all strains (Bonferroni-corrected *P* < 1e-92, hypergeometric test comparing SNPs per total base pairs in each class), likely due to reduced functional constraint in intergenic sequences (Doniger et al. 2008). Novel SNPs show a similar distribution (although with slightly more SNPs in coding regions; Bonferroni-corrected *P* < 1.89e-20 for CRB and MUSH, hypergeometric test comparing SNPs per total base pairs in each class; supplementary table S5, Supplementary Material online), and are also enriched for heterozygous SNPs relative to the background distribution (supplementary table S5, Supplementary Material online; *P* < 1e-4 for all strains, hypergeometric test). This suggests that many of these SNPs represent true SNPs rather than sequencing error, and indicates that our knowledge of the full spectrum of genetic variation in *S. cerevisiae* is still incomplete.

Between 36% and 39% of SNPs detected in coding regions are nonsynonymous relative to the S288c-derived diploid strain DBY8268, indicating that they change the encoded protein sequence (table 1)*.* Interestingly, in CRB, one of the most heterozygous strains, nonsynonymous mutations are more frequent in homozygous SNPs than in heterozygous SNPs (24% vs. 22%, respectively; *P* < 0.0001, χ^2^ test of association), suggesting that these mutations could represent important functional adaptations. Among all three strains, we identified 57 genes with homozygous nonsense mutations, including 14 in CRB, 39 in LEP, and 27 in MUSH (supplementary data set S1, Supplementary Material online). Interestingly, several genes had premature stop codons in multiple strains: Six genes had the same nonsense mutation in all three strains, and 12 genes had the same nonsense mutation in two of the three strains. None of these genes is essential in S288c (Giaever et al. 2002), raising the possibility that they may be undergoing pseudogenization in these strains.

We assessed the nonsynonymous to synonymous substitution rate (*K*_a_/*K*_s_) relative to the S288c lab strain to identify genes with higher than expected rates of coding change (i.e., *K*_a_/*K*_s_ > 1; supplementary table S8, Supplementary Material online). Although this analysis is complicated by short evolutionary timescales (where polymorphisms may be unique to a single lineage and not fixed in the population; Kryazhimskiy and Plotkin 2008), we nonetheless sought to identify genes subject to higher rates of coding polymorphism. Several stress-responsive transcription factors displayed *K*_a_/*K*_s_ > 1 in both CRB (including *GCN4, K*_a_/*K*_s_ = 1.13; *FLO8, K*_a_/*K*_s_ = 1.27; *MOT3, K*_a_/*K*_s_ = 2.54; and *HOT1, K*_a_/*K*_s_ = 1.07; supplementary data set S2*a*, Supplementary Material online) and MUSH (including *MSN4, K*_a_/*K*_s_ = 1.12; *MOT3*, *K*_a_/*K*_s_ = 1.43; and *HOT1*, *K*_a_/*K*_s_ = 1.80; supplementary data set S2*c*, Supplementary Material online). Strikingly, fast-evolving genes in LEP include components of the cell wall integrity *PKC* MAPKKK pathway (including *WSC2*, *K*_a_/*K*_s_ = 1.8; *WSC3*, *K*_a_/*K*_s_ = 1.10, and *BCK1*, *K*_a_/*K*_s_ = 1.26; *P* < 0.003, hypergeometric distribution; supplementary data set S2*b*, Supplementary Material online). Importantly, enrichment of functional categories is not expected, unless many genes involved in the same functional processes have experienced stepwise coevolution (Bullard et al. 2010). These differences may point to mechanisms of increased stress tolerance in these strains (see Discussion).

### Multistress Resistant Strains Have Highly Mosaic Genome Structures

**Fig. 3.—:**
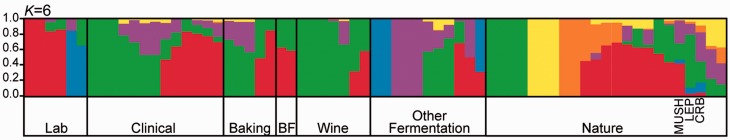
Population structure of 66 *S. cerevisiae* strains. Population structure was inferred using 11,795 evenly distributed SNPs and six ancestral populations, identified as European/wine (green), North American/oak (orange), West African (blue), Sake (purple), and Malaysian (yellow) lineages, as well as various human-associated (red) strains. For each strain indicated on the *x* axis, the height of each colored block represents the proportion of each population assigned to that strain. Labels indicate the source from which each strain was isolated.

We inferred the population structure of 66 strains of *S. cerevisiae*, including CRB, LEP, and MUSH (fig. 3 and supplementary figs. S3 and S4, and table S3, Supplementary Material online). Similar to previous studies, we identified five pure lineages among the strains we analyzed (Malaysian, West African, Sake, North American/oak, and European/wine lineages; Liti et al. 2009; Schacherer et al. 2009). However, the genomes of CRB, LEP, and MUSH are all highly mosaic—portions of their genomes have been inherited from each of the ancestral populations. A large portion of the MUSH genome is similar to those of laboratory strains. By contrast, both the CRB and LEP genomes have segments similar to European/wine and Sake strains. The mosaic nature of these genomes is likely due to infrequent mating among ancestral genomes, leading to novel allele combinations that could underlie the extreme stress tolerance of these strains (see Discussion).

### Non-S288c Genes Are Enriched for Specific Functional Processes

We sought to identify unique genes present in these newly sequenced natural isolates but missing from the common S288c genome reference, through several approaches. First, we mapped the DNA sequencing reads to a composite genome sequence that included approximately 50 kb found in the biofuel strain JAY291 but missing from S288c (Argueso et al. 2009). We found that reads from CRB, but not MUSH or LEP, aligned to the JAY291 sequence. Second, we performed de novo assembly of the short-read sequences and annotated regions that did not align with the S288c lab strain. Finally, we generated RNA-seq data for each strain (see below) and identified transcripts through de novo assembly.

**Fig. 4.—:**
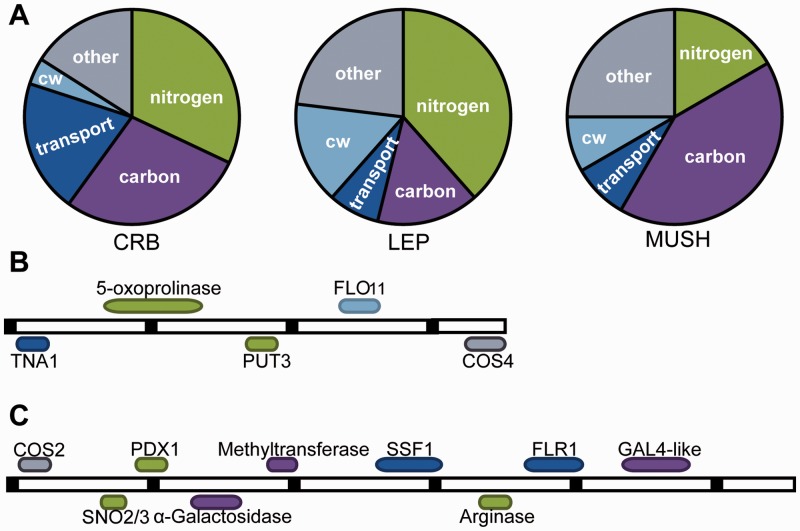
Unique genes in CRB, LEP, and MUSH. A. Functional distribution of 25 non-S288c genes in CRB, 15 non-S288c genes in LEP, and 12 non-S288c genes in MUSH, classified according to predicted GO biological process or molecular function. CW, cell wall; other, unknown or other. (*B* and *C*). Genomic architecture of non-S288c genes in CRB and LEP (*B*) or in CRB and biofuel strain JAY291 (*C*). Black bars are spaced 2 kb apart.

Analysis of non-S288c genome sequence revealed several genes present in our newly sequenced natural isolates, but absent from the reference strain S288c (fig. 4 and supplementary tables S9–S11 Supplementary Material online). We detected 25 genes in 269 kb of non-S288c sequence in CRB, 15 genes in 86 kb of non-S288c sequence in LEP, and 12 genes in 53 kb of non-S288c sequence in MUSH. In all three strains, the majority of genes in non-S288c regions are predicted to be involved in carbon or nitrogen metabolism and transport. LEP showed a preponderance of genes linked to the cell wall and nitrogen catabolism, while in MUSH, non-S288c carbon metabolism genes were most abundant compared with other strains. Both CRB- and LEP-specific regions include portions of a five gene, 14-kb region potentially horizontally acquired from *Zygosaccharomyces bailii* and found in many wine strains (fig. 4*B*; [Galeote et al. 2011; Novo et al. 2009]). CRB also includes a nine gene, 23-kb region present in the biofuels strain JAY291 and other industrial fermentation strains (fig. 4*C*; [Argueso et al. 2009; Babrzadeh et al. 2012]). We also detected sequences similar to several genes that have been pseudogenized or are not present in S288c but are present in other wild strains, including *BIO1, BIO3/6, KHS1, RTM1, AWA1*, and *MPR1* (Goto et al. 1991; Ness and Aigle 1995; Takagi et al. 2000; Shimoi et al. 2002; Hall and Dietrich 2007). Other genes known to vary in copy number, including *MAL* activator genes for maltose fermentation and *SUC* genes for sucrose hydrolysis, were also found in our strains.

In addition to these genes, we identified between 103 and148 transcripts depending on strain that did not match the S288c transcriptome (supplementary table S12, Supplementary Material online). Roughly half of these transcripts matched noncoding regions in the S288c genome, indicating differential transcription potential across strains, in some cases at S288c pseudogenes. Many of the non-S288c transcripts that did not match the genes described above were related to carbon or nitrogen metabolism and transport, further highlighting the prevalence of these functional groups in the variable gene content of the species.

### Significant Transcriptomic Variation in Response to Stress

**Fig. 5.—:**
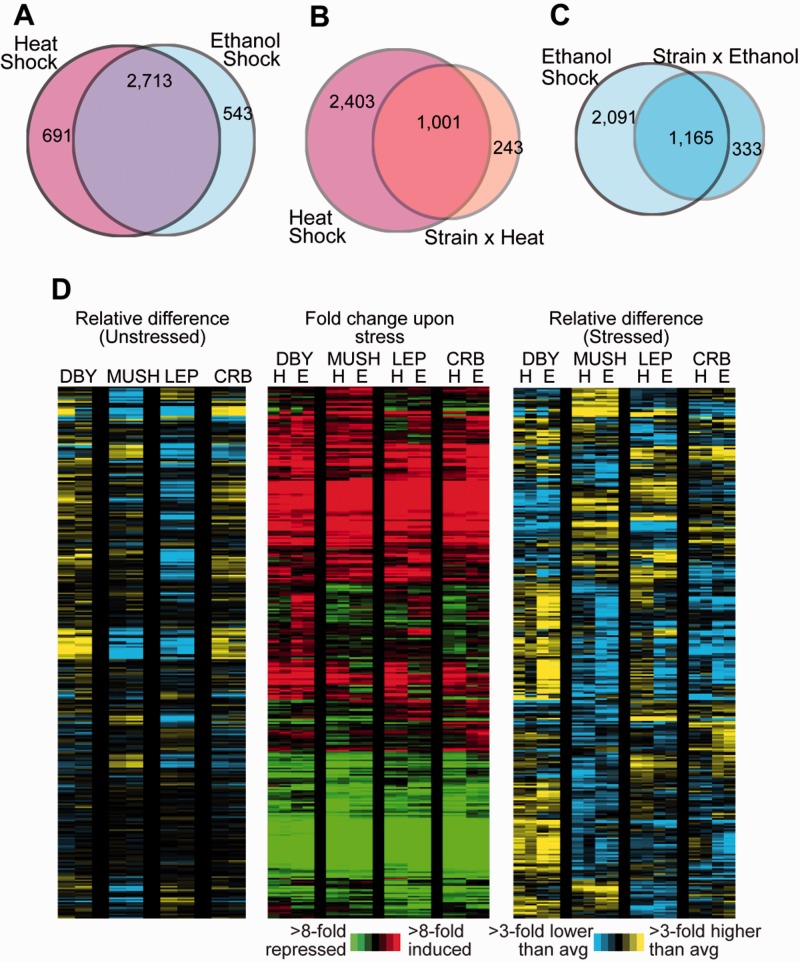
Expression differences across environments and strains. (*A*) Venn diagram representing the number of differentially expressed genes responding to heat or ethanol stress, regardless of strain background. (*B* and *C*) The overlap in heat-responsive genes (independent of strain background, *B*) or ethanol-responsive genes (independent of strain background, *C*) and genes with a strain-by-stress interaction. (*D*) Two hundred forty-eight genes differentially expressed in at least one wild strain. Left: log_2_ expression differences in unstressed strains versus the average of all strains; Middle: log_2_-fold changes in each strain responding to heat (H) or ethanol (E) stress, compared with the strain’s starting expression before addition of stress; Right: Difference in stress-responsive expression in each strain versus the average across all strains. Red/green represents increased/decreased expression in response to each stress; yellow/blue represent higher/lower expression in the denoted strain compared with the average of all strains.

To investigate the mechanisms of stress tolerance, we profiled transcriptome changes in the three natural isolates, along with the diploid S288c-derived strain DBY8268, responding in biological duplicate to two stresses related to biofuels production: A 25–37 °C heat shock or treatment with 5% v/v ethanol. We applied a multifactorial linear model to identify genes differentially expressed in each strain, in response to each environment, and in a manner influenced by strain and environment (so-called “Gene by Environment” interactions). In all, we identified 3,404 and 3,256 genes whose expression was significantly altered in response to heat and/or ethanol treatment, respectively (false discovery rate, FDR < 1%), regardless of strain background (fig. 5*A*). Most of the genes responded to both stresses and included the common environmental stress response that is activated by a wide array of diverse stresses (Gasch et al. 2000). In contrast, 691 and 543 genes responded specifically to heat or ethanol, respectively.

Of the gene expression changes provoked by one or both stresses, we found over 1,962 expression changes that were significantly influenced by genetic background in one or more strains (FDR < 1%), including 478 genes differentially expressed in DBY, 194 in MUSH, 125 in LEP, and 114 in CRB (fig. 5*D*). That the DBY lab strain showed the most strain-specific effects is consistent with prior studies that show this strain is an outlier compared with wild isolates (Kvitek et al. 2008; Lewis et al. 2010; Lewis and Gasch 2012; Hodgins-Davis et al. 2012). The genes with strain-specific effects strongly overlap the genes responsive to heat and ethanol independent of strain background, indicating that much of the genotype-by-environment effects produce quantitative differences in the response. Nonetheless, we also found 243 and 333 genes that showed a heat or ethanol response, respectively, only in specific strains (fig. 5*B* and *C*).

We clustered the strain-specific responses to discover physiological differences in the natural isolates, based on enrichment of GO categories in specific gene clusters. Unlike the other strains, MUSH challenged with ethanol or heat did not strongly induce genes involved hexose transport and amino acid metabolism (Bonferroni-corrected *P* < 0.01, hypergeometric test), perhaps due to reduced impact on these processes. However, MUSH showed greater induction of many stress-activated genes, including those involved in glycogen metabolism. Remarkably, genes with MUSH-specific expression patterns were enriched (Bonferroni-corrected *P* < 0.05, hypergeometric test) for known targets of transcription factors with elevated rates of evolution in MUSH (including Mot3, Hot1, and Msn2 targets, the latter of which strongly overlap with Msn4-regulated genes; table 2; [Berry and Gasch 2008]). CRB showed a unique response to ethanol compared with the other strains, including stronger induction of many stress responsive genes and unique induction of a group of genes involved in aromatic amino acid biosynthesis. CRB-specific expression effects were also enriched (Bonferroni-corrected *P* < 0.05, hypergeometric test) at targets of transcription factors under selection (including Gcn4, Flo8, and Hot1 targets; table 2), indicating that the elevated rates of regulator evolution have downstream consequences.

**Table 2:** Enrichment for Targets of TFs with Evidence for Selection in 217 CRB-Specifically Induced Genes, 311 LEP-Specifically Induced Genes, or 301 MUSH-Specifically Induced Genes

**TF**	***K*_a_**	***K*_s_**	*K*_a_/*K*_s_	***P* value****^a^**	**Number of Targ. in Cluster**	**Number of Targ. in Genome**
**CRB**						
Gcn4	0.0049	0.0043	1.137	6.33e-11	25	118
Flo8	0.0048	0.0037	1.268	1.92e-4	24	216
Hot1	0.0047	0.0044	1.072	0.0404	9	67
**LEP**						
Flo8	0.0059	0.0018	3.288	4.25e-6	33	216
**MUSH**						
Mot3	0.0037	0.0026	1.429	7.64e-4	11	47
Msn2/4	0.0087	0.0078	1.123	0.0457^b^	23	209
Hot1	0.0080	0.0044	1.804	0.0286	11	67

Note.—TFs, transcription factors; Targ., target.

^a^*P* value from Bonferroni-corrected hypergeometric test.

^b^Enrichment for Msn2 targets.

We were particularly interested to see if strain-specific expression differences occurred at genes known to be important for heat or ethanol tolerance in the lab strain. In all strains, the genes with strain-specific responses were enriched for genes identified in screens of the laboratory deletion library as important for ethanol tolerance (*P* < 0.01, hypergeometric test; supplementary table S13, Supplementary Material online). Intriguingly, the affected genes in MUSH included several protein-folding chaperones (including *HSP26, FES1, YDJ1*), whereas genes common to MUSH and CRB included proteins involved in tryptophan and aromatic amino acid biosynthesis (*ARO1, ARO3, TRP2,* and *TRP3*, in one or both strains). These results hint at possible mechanisms of stress tolerance in these strains (see Discussion).

## Discussion

Wild isolates of *S. cerevisiae* represent untapped resources of genetic diversity for genetic engineering and can provide information about the genetic basis of novel phenotypes. Here, we focused on three strains with extreme stress tolerance, which we sought to decipher through genome and transcriptome sequencing. Our results contribute to our understanding of the ecology, evolution, and genotype–phenotype relationships of natural yeast strains.

Differences in the sequence and genome content of the strains studied here suggest disparities in their life histories. All three strains display mosaic genomes, reflecting recent admixture with other distinct lineages through infrequent mating. However, LEP is nearly entirely homozygous, suggesting a clonal lifestyle with little outcrossing to maintain heterozygosity. MUSH and CRB are both highly heterozygous strains, but whereas the MUSH genome is punctuated by regions of LOH, CRB displays little LOH. The latter is surprising, because extensive LOH has been observed in most other sequenced diploid strains of *S. cerevisiae* (Akao et al. 2011; Borneman et al. 2011; Magwene et al. 2011; Babrzadeh et al. 2012). One possibility is that CRB maintains heterozygosity to mask recessive lethal alleles (which may contribute to the low spore viability of this strain) or that the strain may represent a very recent hybrid with some other barrier to spore viability. How mosaicism has influenced the ecological relationships of these strains is unclear. It is notable that the strains sequenced here are generally outliers in terms of stress tolerance, indicating that the phenotypes are not merely due to inheritance of single genes with standing variation. Instead, the reassortment of alleles may have uncovered extreme-stress resistance phenotypes by providing new allelic combinations of relevant gene sets. It is also possible that new mutations specific to these strains contributed to the phenotypes.

Our results expand the known genomic landscape of *S. cerevisiae* as a species. At least 10% of SNPs we identified were previously unknown; their nonrandom genomic distribution suggests that they emerged as new mutations or represent previously unseen minor alleles. A lower bound estimate of 0.4–2% of genome content varies across these strains, corresponding to variation in the presence of 0.2–0.4% of all yeast genes. An interesting theme among these variable genes, as well as transcripts not encoded by the lab-strain genome, is that many are related to carbon and nitrogen metabolism as well as transport. Differences in carbon utilization, nitrogen/amino acid metabolism, and transporter functions have been previously found to vary within and across yeast species, in a variety of studies (Townsend 2003; Kvitek et al. 2008; Hittinger et al. 2010; Wenger et al. 2010; Will et al. 2010; Chang and Leu 2011; Gutierrez et al. 2013; Opulente et al. 2013), raising the possibility that these processes are highly variable in nature.

We also uncovered a striking level of nonsynonymous coding differences, in some cases reflecting nonsense alleles and in others revealing elevated rates of change that can be a signature of selection. The significant differences across this relatively small set of strains underscore the level of genetic diversity in the species. Although many of these differences may be (nearly) neutral, others may contribute to phenotypic differences across strains. Along these lines, it is especially notable that half of the environment-responsive transcript changes we observed showed strain-specific effects. Furthermore, the transcripts expressed in a strain-specific manner were enriched for targets of transcription factors putatively under positive selection in individual strains (*K*_a_/*K*_s_ > 1). Thus, genotype-by-environment interactions are prevalent and likely affect many different yeast phenotypes.

The genomic differences uncovered here provide clues to the potential mechanisms of stress tolerance. The ethanol-tolerant LEP strain carries a preponderance of non-S288c genes related to the cell wall (fig. 4) and displays elevated rates of coding-sequence changes in genes in the *PKC* cell wall-integrity signaling pathway. Together, these data raise the possibility that cell wall differences have been positively selected in the LEP background. Given that the cell surface is a prime target of ethanol stress (van Voorst et al. 2006; Teixeira et al. 2009), and cell wall differences could contribute to the LEP ethanol resistance trait.

The heat-resistant MUSH strain displays several unique features related to carbon metabolism—previously shown to impact thermotolerance (Gibney et al. 2013)—including an enrichment of non-S288c genes linked to carbon response and differences in expression of carbon metabolism genes. MUSH also shows elevated sequence changes in stress-activated transcription factors, including the multistress activated Msn4, and hyper-activation of Msn2/Msn4 targets including protein-folding chaperones required for heat survival.

Finally, the multistress resistant CRB strain displays unique induction of genes involved in aromatic amino acid metabolism, which were previously shown important for ethanol tolerance, perhaps due to alterations in membrane fluidity (Hirasawa et al. 2007; Yoshikawa et al. 2009). Notably, lab strains lacking these genes emerge as sensitive to ACSH in several high-throughput screens (manuscript in preparation), suggesting that the unique expression response in CRB may also contribute to ACSH resistance.

The level of phenotypic variation in natural and industrial *S. cerevisiae* strains is only beginning to emerge, thanks to high-throughput screening efforts. An important area of ongoing work is to determine the causal genetic differences that underlie those traits and their evolutionary histories. Nonetheless, natural variation in wild strains provides an excellent starting point for future dissection of stress-resistance mechanisms as well as engineering for bioproduct formation.

## Supplementary Material

Supplementary figures S1–S6, tables S1–S13, notes S1 and S2, data set S1–S7, methods, and references are available at *Genome Biology and Evolution* online (http://www.gbe.oxfordjournals.org/).

Supplementary Data
