# Supplementary material for: Comparative Genomics of Saccharomyces cerevisiae Natural Isolates for Bioenergy Production
Source: Genome Biol Evol. 2014 Sep 5;6(9):2557–66. doi: 10.1093/gbe/evu199 (PMC4202335; doi:10.1093/gbe/evu199)
Supplement: Supplementary Data [file supp_6_9_2557__index.html]

Comparative genomics of Saccharomyces cerevisiae natural isolates for bioenergy production — Comparative Genomics of Saccharomyces cerevisiae Natural Isolates for Bioenergy Production — Supplementary Data 

# Comparative Genomics of *Saccharomyces cerevisiae* Natural Isolates for Bioenergy Production

## Supplementary Data

files

**Files in this Data Supplement:**

- Supplementary Data - zip file
