# Supplementary material for: Comparative Genomics of Saccharomyces cerevisiae Natural Isolates for Bioenergy Production
Source: Genome Biol Evol. 2014 Sep 5;6(9):2557–66. doi: 10.1093/gbe/evu199 (PMC4202335; doi:10.1093/gbe/evu199)
Supplement: Supplementary Data [file supp_evu199_suppl_data.zip › WohlbachSupplementaryInformation-2014-GBE_Revised.pdf]

Supplementary Information Appendix for:

Comparative genomics of *Saccharomyces cerevisiae* natural isolates for bioenergy production

Dana J. Wohlbach [1,2,4], Nikolay Rovinskiy [1,2], Jeffrey A. Lewis [1,2,5], Maria Sardi [1,2], Wendy S. Schackwitz [3], Joel A. Martin [3], Shweta Deshpande [3], Christopher G. Daum [3], Anna Lipzen [3], Trey K. Sato [2], Audrey P. Gasch [1,2,*]

[1] Laboratory of Genetics; University of Wisconsin; Madison, Wisconsin 53706

[2] DOE Great Lakes Bioenergy Research Center; University of Wisconsin; Madison, Wisconsin 53726

[3] US Department of Energy Joint Genome Institute; Walnut Creek, California 94598

[4] Current address: Biology Department; Dickinson College; Carlisle, Pennsylvania 17013

[5] Current address: Department of Biological Sciences; University of Arkansas; Fayetteville, AR 72701

* Author for Correspondence: Audrey P. Gasch, Laboratory of Genetics, University of Wisconsin-Madison; phone: 608-265-0859; agasch@wisc.edu

SUPPLEMENTARY NOTES

Note S1: Varying levels and patterns of heterozygosity in multi-stress resistant strains. We observed six large regions of LOH in MUSH: in the right arms of chromosome II, VII, XII, and XV, and in the left arms of chromosomes II and IX (Table S4). These regions of LOH vary in size from ~25 kb to ~460 kb, and in all cases continue to the ends of chromosomes. Interestingly, many of the MUSH regions of LOH overlap with regions of LOH found in other heterozygous strains (Babrzadeh, et al., 2012; Magwene, et al., 2011).

Note S2: Extensive copy number variation in multi-stress resistant strains. We assessed copy number variation (CNV) in CRB, LEP, and MUSH relative to the diploid reference lab strain DBY8628 using array-based comparative genomic hybridizations in biological duplicate. Consistent with previous studies (Bergström, et al., 2014; Dunn, et al., 2012; Kvitek, et al., 2008), we observed numerous amplifications and deletions in all of the natural isolates relative to the reference strain, most notably in subtelomeric regions (Figure S4 and Dataset S3).

Using aCGH, we also observed large amplified regions found near the ends of every chromosome in CRB, a finding supported by sequence-read depth of coverage (DOC; Figure S5). Although this pattern of sub-telomeric amplification was observed for multiple different DNA preparations, performed side-by-side with reference strains, and using two different methodologies (aCGH and DOC), we were concerned that the pattern was artifactual. To address this issue, we performed physical separation of chromosomes with pulsed-field gel electrophoresis (PFGE; Figure S6). PFGE does not support the large-scale amplifications observed by aCGH in CRB; however, this technique does indicate karyotype differences for CRB, and possibly MUSH.

Sequence-read DOC also suggested that CRB is triploid at Chr I. However, we note that assessing CNV with short-read sequencing presents unique challenges, particularly for sub-telomeric regions which show poor structural conservation (Bergström, et al., 2014).

SUPPLEMENTARY METHODS

Read mapping and SNP calling for Genome Sequencing. Reads were trimmed at the 3' end when base quality fell below Q20 using Trimmomatic (v0.30; (Bolger, et al., 2014)). Reads were mapped to a composite *S. cerevisiae* reference genome, including strain S288c (NCBI RefSeq, February 2011) plus sequence missing from S288c but identified in biofuels strain JAY291 (Argueso, et al., 2009). Read mapping was performed with Burrows-Wheeler Alignment (BWA, v1.2.2; (Li, et al., 2009)) using default parameters except the fraction of missing alignments threshold was 0.08 (-n in "bwa aln").

SNP and indel detection was performed with the Genome Analysis Toolkit (GATK, v2.7; (McKenna, et al., 2010)), following their "best-practice" variant calling workflow for calling variants with the HaplotypeCaller (www.broadinstitute.org/gatk/). Duplicate reads were marked, followed by base quality recalibration using a dbSNP designed for *S. cerevisiae* (see Population genomics analysis). To minimize false positive SNP calls, we applied variant quality score recalibration (VQSR) at the 90% threshold. To minimize false positive indel calls, stringent parameters were used to filter variants on the basis of variant confidence by depth (QD < 2.0), strand bias (FS > 200), and distance from the end of the read (ReadPosRankSum < -20.0). Custom perl scripts were used to further analyze variant output.

Genome Sequencing *de novo* Assembly. *de novo* genome assembly was performed using String Graph Assembler (SGA, v0.9.19; (Simpson, et al., 2012)), with a minimum k-mer coverage of 2 (-x in "sga filter") and a minimum length of a contig for inclusion in a scaffold of 200 bp (-m in "sga scaffold"). We also tuned the correction k-mer value (-k in "sga correct") and the minimum overlap (-m in "sga fm-merge") for each genome (Table S13). Contigs generated from the *de novo* assembly were aligned to the S288c genome using the nucmer algorithm in MUMmer (v3.0; (Kurtz, et al., 2004)), and novel sequence not present in S288c was identified using custom perl scripts. Gene prediction on novel sequence was performed using GlimmerHMM (v3.0.2; (Majoros, et al., 2004)) trained on *S. cerevisiae* transcripts. Genome sequencing data for each strain are available through their US DOE Joint Genome Institute genome portals (http://jgi.doe.gov/).

RNA-Seq *de novo* Assembly and Counting. Read counts per gene were summarized using HTSeq (v0.5.4p3; http://www-huber.embl.de/users/anders/HTSeq/). The general linearized model used for differential expression analysis identifies genes whose expression changes in response to each condition regardless of strain background, in each strain regardless of treatment, and in a manner where strain and condition interact (*i.e.* genes with genotype-by-environment interaction). To identify transcripts *de novo*, paired-end fastq files containing the sequencing results were subjected to Trinity (Grabherr, et al.,

2011). Identified transcripts were subjected to BLAST (v2.2.28+; (Camacho, et al., 2009)) searches against the S288c transcriptome. Sequences with significant hits (E-value < 10^{-5}) were removed, and redundant transcripts from each strain were collapsed, taking the longest transcript as a representative. Transcripts were compared to the S288c transcriptome using BLAST (v2.2.28+; (Camacho, et al., 2009)). To identify homology to other known sequences, the remaining transcripts were subjected to blast against the NCBI nucleotide collection or blastx against NCBI non-redundant protein sequence database. Resulting transcripts are available in Dataset S5.

For other expression analysis, genes with fewer than ten mapped reads in at least one experiment were removed from subsequent analysis. The RNA-Seq data were converted to reads per kilobase of gene per million mapped reads (Mortazavi, et al., 2008) and the $\log_2$ fold-change in expression after treatment was taken for subsequent analysis. In some cases, the $\log_2$ expression difference between each strain and the average of all strains was also analyzed. These data were subjected to model-based clustering using the *mclust()* R package (Fraley, et al., 2012) with the VII model and k = 100 clusters. Highly similar clusters were collapsed using custom scripts that considered 1) cluster centroid similarity and 2) heterogeneity in potentially merged clusters. Cluster centroids were arranged by hierarchically clustered based on the Pearson product-moment correlation coefficient. Cluster heterogeneity was scored as standard deviation of gene expression covariances within the cluster. Clusters identified by mclust were collapsed only if the Pearson product-moment correlation coefficient of their centroids was > 0.8 and heterogeneity in merged cluster was ≤ 0.5. The process was performed iteratively until stable. Results from edgeR and processed expression data are available in Datasets S6 and S7, respectively.

Pulsed-Field Gel Electrophoresis (PFGE). Agarose-embedded chromosomal DNA was prepared using InCert agarose (Lonza Rockland, Inc.) as previously described (Lai, et al., 1989). Chromosomes were separated using a CHEF-DR III system (BioRad). Gels were run for 24 h, with a 60 s pulse time at a 120˚ angle. Migrations were performed at 14˚C with a constant voltage of 6 V/cm.

SUPPLEMENTARY REFERENCES

Akao T, *et al*. 2011. Whole-genome sequencing of sake yeast *Saccharomyces cerevisiae* Kyokai no. 7. DNA Res. 18:423-434.

Argueso JL, *et al*. 2009. Genome structure of a *Saccharomyces cerevisiae* strain widely used in bioethanol production. Genome Res. 19:2258-2270.

Babrzadeh F, *et al*. 2012. Whole-genome sequencing of the efficient industrial fuel-ethanol fermentative *Saccharomyces cerevisiae* strain CAT-1. Mol Genet Genomics. 287:485-494.

Bergström A, *et al*. 2014. A high-definition view of functional genetic variation from natural yeast genomes. Mol Biol Evol. 31:872-888.

Bolger AM, Lohse M and Usadel B. 2014. Trimmomatic: a flexible trimmer for Illumina sequence data. Bioinformatics.

Borneman AR, *et al*. 2011. Whole-genome comparison reveals novel genetic elements that characterize the genome of industrial strains of *Saccharomyces cerevisiae*. PLoS Genet. 7:e1001287.

Camacho C, *et al*. 2009. BLAST+: architecture and applications. BMC Bioinformatics. 10:421-429.

Doniger SW, *et al*. 2008. A catalog of neutral and deleterious polymorphism in yeast. PLoS Genet. 4:e1000183.

Dunn B, *et al*. 2012. Analysis of the *Saccharomyces cerevisiae* pan-genome reveals a pool of copy number variants distributed in diverse yeast strains from differing industrial environments. Genome Res. 22:908-924.

Fraley C, Raftery AE, Murphy TB and Scrucca L. mclust version 4 for R: Normal mixture modeling for model-based clustering, classification, and density estimation. In.; 2012.

Gibney PA, *et al*. 2013. Yeast metabolic and signaling genes are required for heat-shock survival and have little overlap with the heat-induced genes. Proc Natl Acad Sci USA. 110:E4393-4402.

Grabherr MG, *et al*. 2011. Full-length transcriptome assembly from RNA-Seq data without a reference genome. Nat Biotechnol. 29:644-652.

Kubota S, *et al*. 2004. Effect of ethanol on cell growth of budding yeast: genes that are important for cell growth in the presence of ethanol. Biosci Biotech Bioch. 68:968-972.

Kurtz S, *et al*. 2004. Versatile and open software for comparing large genomes. Genome Biol. 5:R12.

Kvitek DJ, Will JL and Gasch AP. 2008. Variations in stress sensitivity and genomic expression in diverse *S. cerevisiae* isolates. PLoS Genet. 4:e1000223.

Lai E*, et al*. 1989. Pulsed field gel electrophoresis. BioTechniques. 7:34-42.

Li H and Durbin R. 2009. Fast and accurate short read alignment with Burrows-Wheeler transform. Bioinformatics. 25:1754-1760.

Liti G*, et al*. 2009. Population genomics of domestic and wild yeasts. Nature. 458:337-341.

Magwene PM*, et al*. 2011. Outcrossing, mitotic recombination, and life-history trade-offs shape genome evolution in *Saccharomyces cerevisiae*. Proc Natl Acad Sci USA. 108:1987-1992.

Majoros WH, Pertea M and Salzberg SL. 2004. TigrScan and GlimmerHMM: two open source *ab initio* eukaryotic gene-finders. Bioinformatics. 20:2878-2879.

McKenna A*, et al*. 2010. The Genome Analysis Toolkit: a MapReduce framework for analyzing next-generation DNA sequencing data. Genome Res. 20:1297-1303.

Mortazavi A*, et al*. 2008. Mapping and quantifying mammalian transcriptomes by RNA-Seq. Nat Methods. 5:621-628.

Simpson JT and Durbin R. 2012. Efficient *de novo* assembly of large genomes using compressed data structures. Genome Res. 22:549-556.

Teixeira MC*, et al*. 2009. Genome-wide identification of *Saccharomyces cerevisiae* genes required for maximal tolerance to ethanol. Appl Environ Microbiol. 75:5761-5772.

van Voorst F*, et al*. 2006. Genome-wide identification of genes required for growth of *Saccharomyces cerevisiae* under ethanol stress. Yeast. 23:351-359.

Wei W*, et al*. 2007. Genome sequencing and comparative analysis of *Saccharomyces cerevisiae* strain YJM789. Proc Natl Acad Sci USA. 104:12825-12830.

Yoshikawa K*, et al*. 2009. Comprehensive phenotypic analysis for identification of genes affecting growth under ethanol stress in *Saccharomyces cerevisiae*. FEMS Yeast Res. 9:32-44.

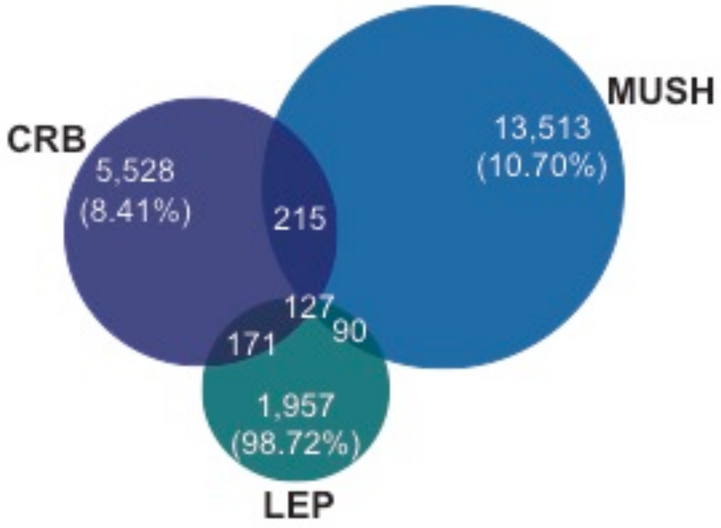

Figure S1. Distribution of novel SNPs detected in this study. The 21,601 SNPs represented here were not detected in any other fully sequenced strain of *S. cerevisiae*. Of these, 603 were detected in more than one of our strains; only 127 unique SNPs were commonly identified in all three of our strains. Numbers in parentheses represent the proportion of SNPs that are homozygous for a given strain. See also Table S1.

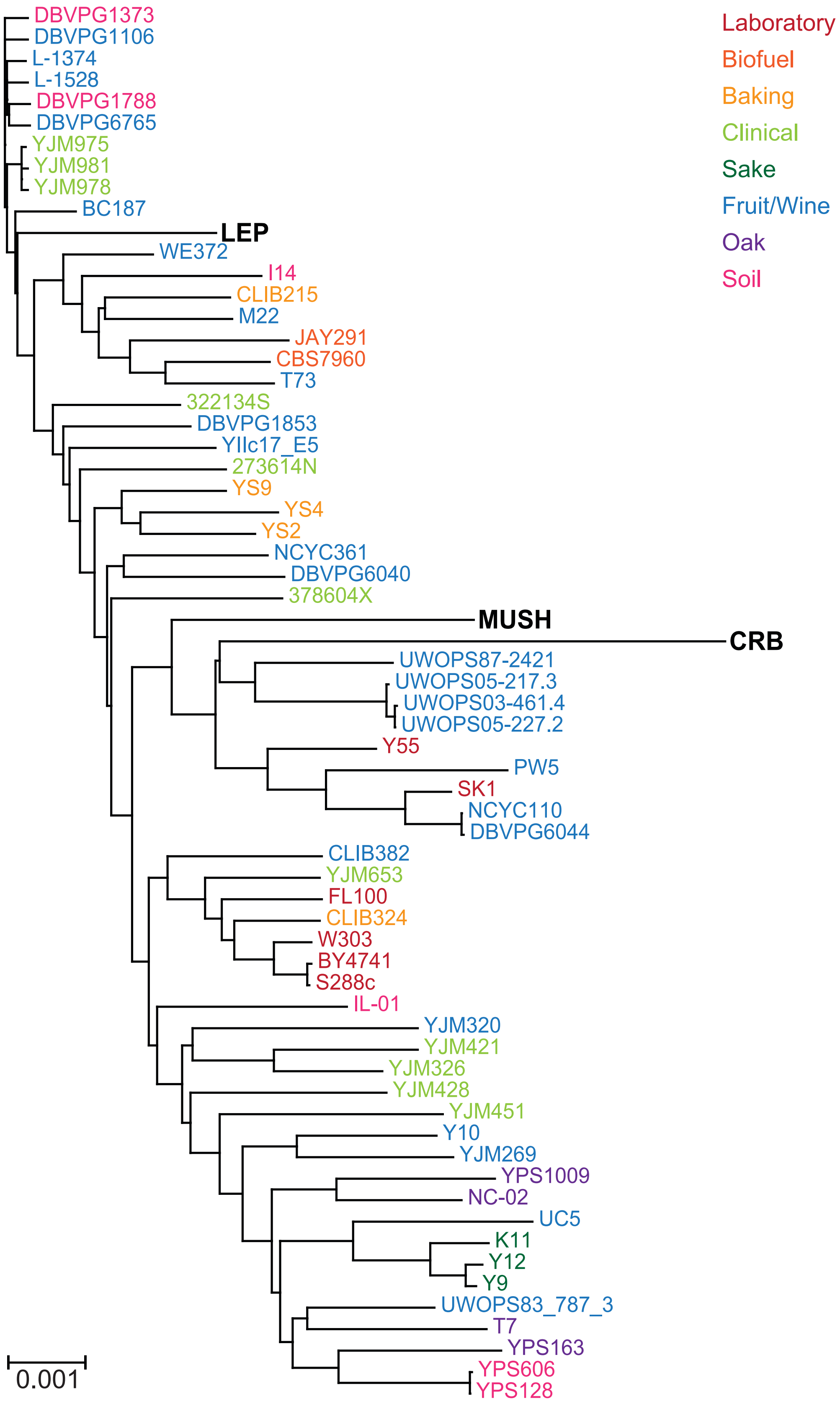

Figure S2. Neighbor-joining phylogeny of 66 *S. cerevisiae* strains. Strains are color-coded according to the niche from which they were isolated. Scale bar represents nucleotide substitutions per site.

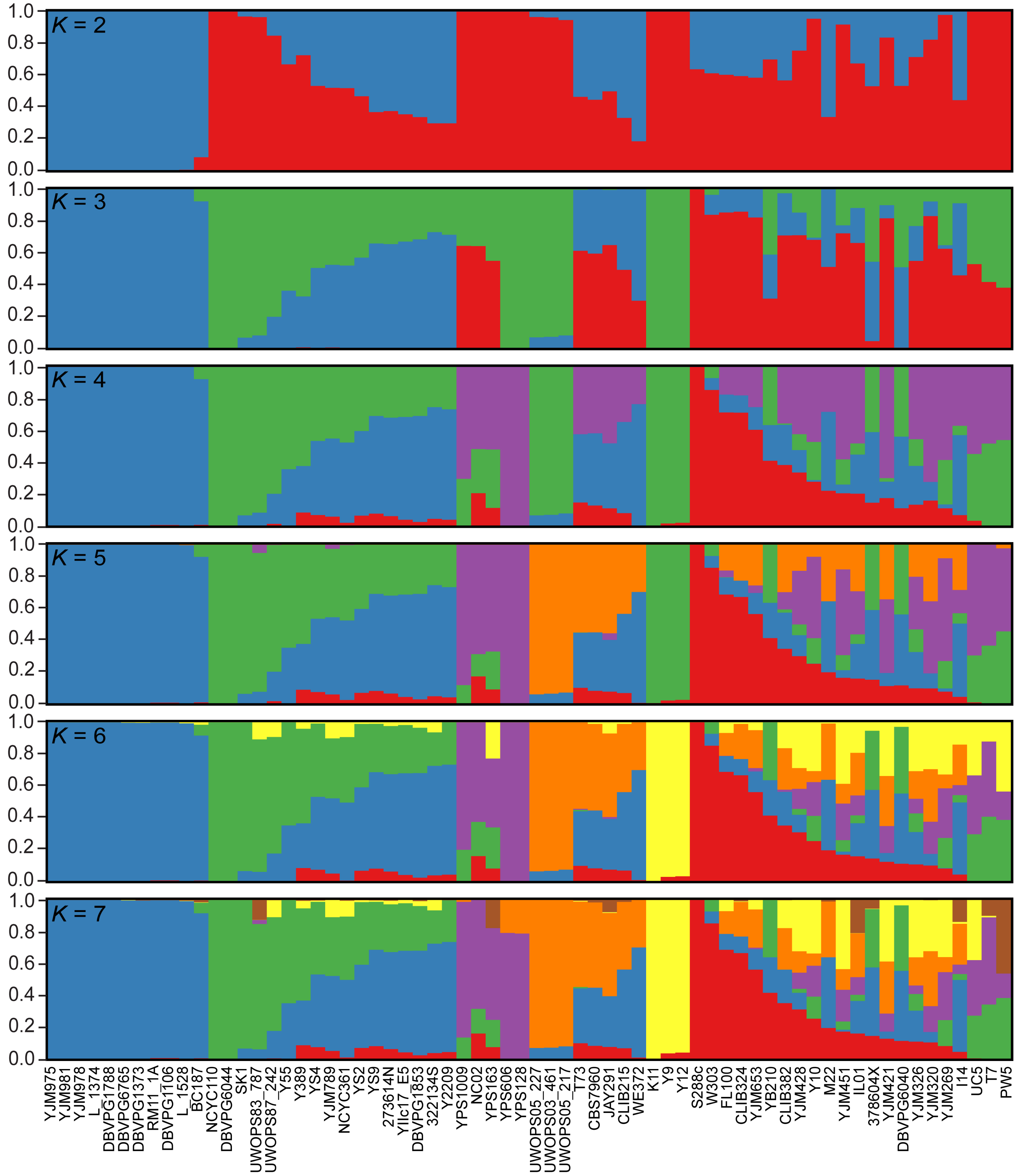

Figure S3. Population structure of 66 *S. cerevisiae* strains for *K* = 2 through 7. Population structure was inferred using 11,795 evenly distributed SNPs and two to seven ancestral populations, under the linkage model for 50,000 iterations, after a burn-in of 20,000 to 40,000 iterations. We estimated the posterior probability for each K by assuming a uniform prior on $K = \{1, \ldots, 7\}$, and determined that $K = 6$ captures most of the structure of the data, including the European/wine (blue), West African (green), North American/oak (purple), Malaysian (orange), Sake (yellow) lineages and human-associated (red) strains. For each strain indicated on the x-axis, the height of each colored block represents the proportion of each population assigned to that strain.

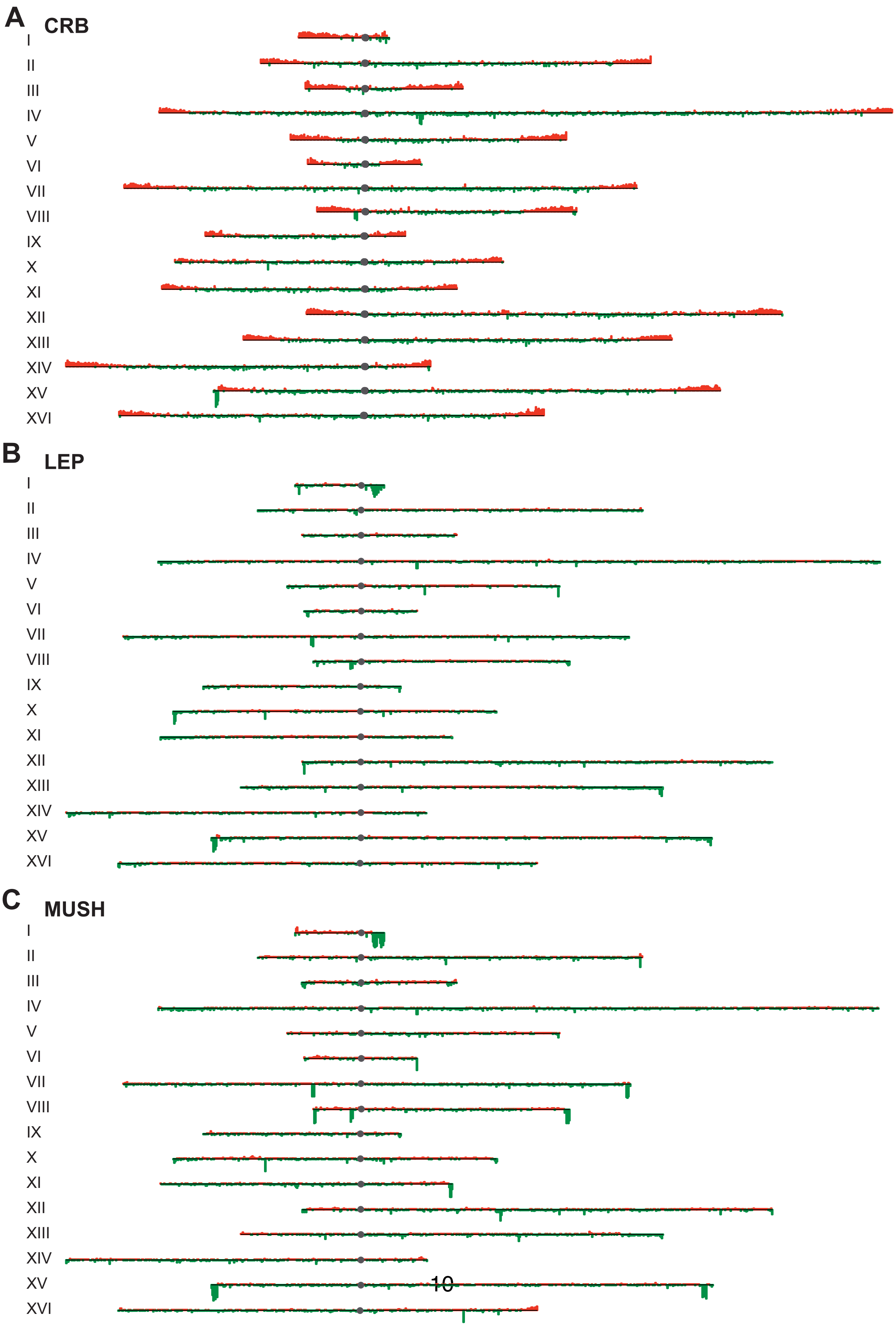
A
CRB
B
LEP
C
MUSH
I
II
III
IV
V
VI
VII
VIII
IX
X
XI
XII
XIII
XIV
XV
XVI

D

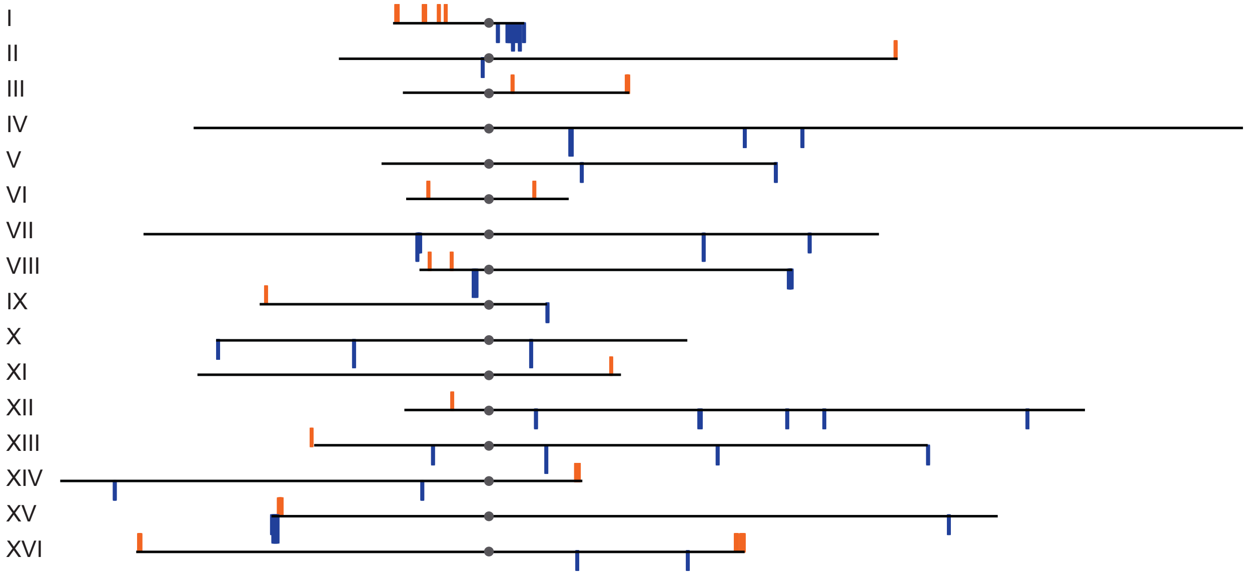

Figure S4. Array-based comparative genomic hybridization (aCGH) reveals sub-telomeric amplifications and deletions. A-C. Genomic DNA samples from CRB (**A**), LEP (**B**), and MUSH (**C**) were competitively hybridized against the S288c-derived reference strain DBY8268 to whole-genome arrays. Data are organized by chromosome, aligned at the centromere (grey circles). Each bar depicts an individual gene in the reference strain: red bars above the x-axis represent increased signal in CRB relative to the reference; green bars below the x-axis represent decreased signal in CRB relative to the reference. Data represent the mean $\log_2$ (CRB signal / DBY8268 signal) of biological replicates (n = 2). All y-axes scale from 2.5 to -2.5. **D.** Common amplifications (orange bars above the x-axis) and deletions (blue bars below the x-axis) among more than one newly sequenced strain are depicted. Bars represent the total number of strains sharing a given amplification or deletion (2 or 3).

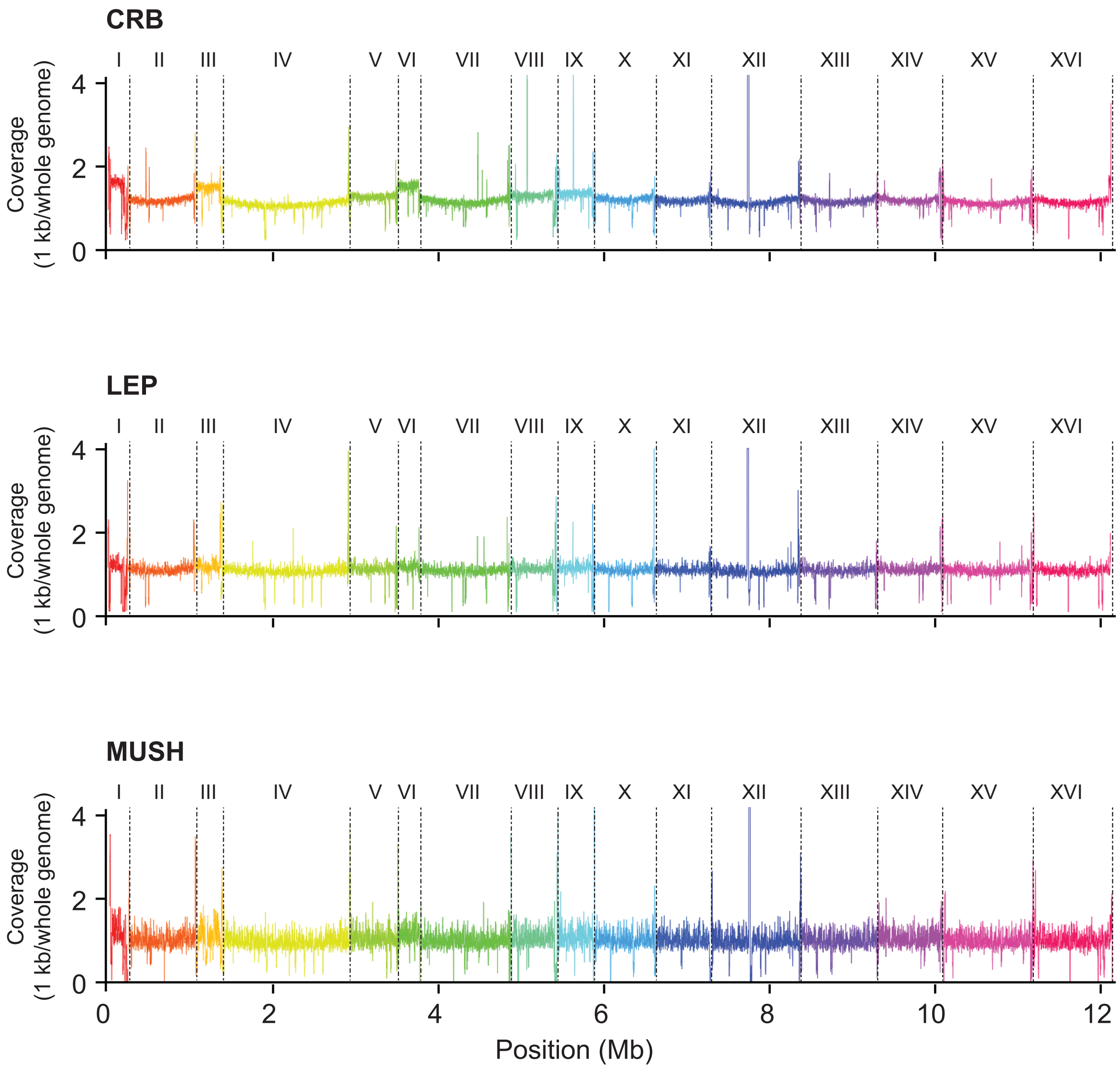

Figure S5. Sequencing read depth of coverage (DOC) reveals aneuploidy in CRB. The median DOC for all reads in sliding 1 kb windows with a 100 bp step size is plotted relative to the total median genomic coverage of each strain. Individual chromosomes are demarked by color and dashed vertical lines. For a diploid strain, a relative DOC of 1 indicates the chromosome is present at 1X (i.e. two copies); a relative DOC of ~1.5 suggests there are 3 copies of the chromosome.

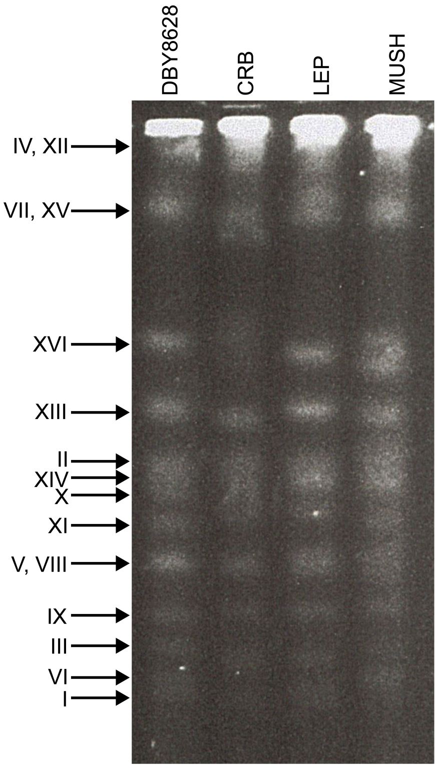

Figure S6. Chromosomal separation by pulsed-field gel electrophoresis (PFGE). Chromosomes are labeled according to known sizes of the S288c derivative DBY8628.

Table S1. Total number indels detected among the three newly-sequenced strains.

Strain	Insertions [a]	Deletions [a]	Source
CRB	3,475	3,874	Spoiled banana, Costa Rica
LEP	2,282	2,800	*Lepidopterous,* San Jacinto, California
MUSH	6,322	3,779	Mushrooms

[a] Indels <100 bp

Table S2. Distribution of novel SNPs across the three newly-sequenced strains. [a]

Strain	Intergenic [b]	Heterozygous [c]	Total
CRB	1,960 (30.57%)	5,063 (91.59%)	5,528
LEP	591 (30.20%)	25 (1.28%)	1,957
MUSH	3,902 (28.88%)	12,067 (89.30%)	13,513

[a] See Figure S1 for overlap between strains.
[b,c] Numbers in parentheses represent the proportion of the total for each strain that are categorized as indicated.

Table S3. Comparisons of heterozygosity in sequenced diploid strains of *S. cerevisiae*.

Strain	Origin	Homozygous SNPs	Heterozygous SNPs	Reference
MUSH	mushroom	25,538	52,516	This work
CRB	banana	25,383	47,138	This work
YJM223	clinical	25,683	37,148	(Magwene, et al., 2011)
CAT-1	bioethanol	36,902	34,503	(Babrzadeh, et al., 2012)
YJM128	clinical	31,406	33,457	(Magwene, et al., 2011)
FostersB	ale	23,125	33,071	(Borneman, et al., 2011)
FostersO	ale	25,802	27,215	(Borneman, et al., 2011)
EM93	fig	5,692	24,420	(Magwene, et al., 2011)
YJM311	clinical	27,623	23,852	(Magwene, et al., 2011)
YJM309	clinical	37,747	22,987	(Magwene, et al., 2011)
YJM308	clinical	28,031	22,229	(Magwene, et al., 2011)
QA23	wine	4,913	18,861	(Borneman, et al., 2011)
Vin13	wine	3,544	15,216	(Borneman, et al., 2011)
VL3	wine	5,108	9,904	(Borneman, et al., 2011)
PMY112	vineyard	37,402	6,480	(Magwene, et al., 2011)
PMY110	vineyard	36,723	6,045	(Magwene, et al., 2011)
PMY093	grape	51,371	4,086	(Magwene, et al., 2011)
K7	sake	66,378	1,347	(Akao, et al., 2011)
AWRI796	wine	8,996	1,041	(Borneman, et al., 2011)
YJM222	clinical	48,164	728	(Magwene, et al., 2011)
YPS670	oak	57,766	551	(Magwene, et al., 2011)
∑1278b	laboratory	28,110	337	(Magwene, et al., 2011)
LEP	leaves	44,149	267	This work

Table S4. Regions of loss of heterozygosity (LOH) in MUSH.

Chr.	Arm	LOH Pos. (bp)	Size (kb)	In Other Strains [a]
II	left	181,959	181.96	YJM308
II	right	395,900	417.28	YJM309
VII	right	1,066,657	24.29	
IX	left	105,430	105.43	
XII	right	614,742	463.44	EM93, YJM128, YJM311, CAT-1
XV	right	850,844	240.45	PMY110, CAT-1

[a] Data for LOH regions in other strains from (Magwene, et al., 2011) and (Babrzadeh, et al., 2012).

Table S5. K_a/K_s summary statistics.

	K_a		K_s		K_a/K_s		
	**Median**	**Mean (SD)**	**Median**	**Mean (SD)**	**Median**	**Mean (SD)**	**Max**
**CRB**	0.00208	0.00269 (0.00290)	0.01572	0.01764 (0.01113)	0.13162	0.19861 (0.25561)	3.01601
**LEP**	0.00142	0.00202 (0.00242)	0.01094	0.01324 (0.01015)	0.12871	0.21257 (0.29241)	3.84476
**MUSH**	0.00265	0.00324 (0.00307)	0.01706	0.01917 (0.01214)	0.15529	0.23209 (0.27901)	3.56706

Table S6. Sequenced strains used for population genomics comparisons in this study.

Strain	Classification	Location	Date	Source	Sequence Reference [a,b]
FL100	laboratory	Alsace, France	pre-1968	Laboratory	*S. cerevisiae* Strain Project
S288c	laboratory	Merced, California, USA	1938	Rotting fig	SGD
SK1	laboratory	USA	pre-1974	Soil	(Liti, et al., 2009)
W303	laboratory	NA	NA	NA	(Liti, et al., 2009)
Y55	laboratory	France	1930-60	Grape	(Liti, et al., 2009)
CLIB215	baking	New Zealand	1994	Baker's strain	*S. cerevisiae* Strain Project
CLIB324	baking	Saigon, Vietnam	1996	Baker's strain	*S. cerevisiae* Strain Project
YS2	baking	Australia	NA	Baker's strain	(Liti, et al., 2009)
YS4	baking	Netherlands	1975	Baker's strain	(Liti, et al., 2009)
YS9	baking	Singapore	NA	Baker's strain	(Liti, et al., 2009)
CBS7960	biofuel	Sao Paulo, Brazil	NA	Factory that produces ethanol from cane-sugar syrup	*S. cerevisiae* Strain Project
JAY291	biofuel	Brazil	NA	Bioethanol distillery	(Argueso, et al., 2009)
273614N	clinical	Newcastle, United Kingdom	NA	Fecal isolate from Royal Victoria Infirmary	(Liti, et al., 2009)
322134S	clinical	Newcastle, United Kingdom	NA	Throat-sputum isolate from Royal Victoria Infirmary	(Liti, et al., 2009)
378604X	clinical	Newcastle, United Kingdom	NA	Sputum isolate from Royal Victoria Infirmary	(Liti, et al., 2009)
YJM320	clinical	USA	pre-1994	Blood	*S. cerevisiae* Strain Project
YJM326	clinical	USA	pre-1994	Clinical isolate	*S. cerevisiae* Strain Project
YJM421	clinical	USA	pre-1994	Ascites fluid	*S. cerevisiae* Strain Project
YJM428	clinical	USA	pre-1994	Paracentesis fluid	*S. cerevisiae* Strain Project
YJM451	clinical	Europe	pre-1994	Clinical isolate	*S. cerevisiae* Strain Project
YJM653	clinical	Unknown	NA	Broncho-alveolar lavage	*S. cerevisiae* Strain Project
YJM789	clinical	USA	NA	Isolated from AIDS patient with pneumonia	(Wei, et al., 2007)
YJM975	clinical	Italy	1994-96	Isolated from vagina of patient suffering from vaginitis	(Liti, et al., 2009)
YJM978	clinical	Italy	1994-96	Isolated from vagina of patient suffering from vaginitis	(Liti, et al., 2009)
YJM981	clinical	Italy	1994-96	Isolated from vagina of patient suffering from vaginitis	(Liti, et al., 2009)
DBVPG1106	nature	Australia	1947	grapes	(Liti, et al., 2009)

Strain (cont.)	Classification	Location	Date	Source	Sequence Reference [a,b]
DBVPG1373	nature	Netherlands	1952	Soil	(Liti, et al., 2009)
DBVPG1788	nature	Turku, Finland	1957	Soil	(Liti, et al., 2009)
DBVPG6765	nature	Indonesia	NA	Lici fruit	(Liti, et al., 2009)
I14	nature	Petrina, Italy	2002	Soil	(Liti, et al., 2009)
IL-01	nature	Cahokia, Illinois, USA	2003	Soil	*S. cerevisiae* Strain Project
M22	nature	Italy	NA	Vineyard	(Doniger, et al., 2008)
UWOPS03-461.4	nature	Telok Senangin, Malaysia	2005	Nectar, Bertram palm	(Liti, et al., 2009)
UWOPS05-217.3	nature	Telok Senangin, Malaysia	2005	Nectar, Bertram palm	(Liti, et al., 2009)
UWOPS05-227.2	nature	Telok Senangin, Malaysia	2005	*Trigona* spp. (stingless bee) collected near Bertam palm flower	(Liti, et al., 2009)
UWOPS83-787.3	nature	Great Inagua Island, Bahamas	1983	Fruit, *Opuntia stricta*	(Liti, et al., 2009)
UWOPS87-2421	nature	Puhelu Road Maui, Hawaii, USA	1987	Cladode, *Opuntia megacantha*	(Liti, et al., 2009)
Y10	nature	Philippines	pre-1973	Coconut	*S. cerevisiae* Strain Project
LEP (Y-2209)	nature	San Jacinto, California, USA	NA	Leaves from *Lepidopterous*	This work
MUSH (Y-389)	nature	Unknown	NA	Mushrooms	This work
CRB (YB-210)	nature	Costa Rica	NA	Spoiled banana	This work
YJM269	nature	Portugal	1954	Blauer Portugieser grapes	*S. cerevisiae* Strain Project
YPS128	oak	Pennsylvania	1999	Soil beneath *Quercus alba*	(Liti, et al., 2009)
YPS606	oak	Pennsylvania	1999	Bark of *Quercus rubra*	(Liti, et al., 2009)
NC-02	oak	Smoky Mountains, North Carolina, USA	2003	Oak tree exudate	*S. cerevisiae* Strain Project
T7	oak	Babler State Park, Missouri, USA	2003	Oak tree exudate	*S. cerevisiae* Strain Project
YPS1009	oak	Mettler Woods, New Jersey, USA	2000	Oak tree exudate	*S. cerevisiae* Strain Project
YPS163	oak	Pennsylvania, USA	1999	Oak tree exudate	(Doniger, et al., 2008)
CLIB382	other fermentation	Ireland	pre-1952	Super-attenuated beer from brewery	*S. cerevisiae* Strain Project
DBVPG1853	other fermentation	Ethiopia	1959	White tecc	(Liti, et al., 2009)
DBVPG6040	other fermentation	Netherlands	1947	Fermenting fruit juice	(Liti, et al., 2009)
DBVPG6044	other fermentation	West Africa	1925	Bili wine from *Osbeckia grandiflora*	(Liti, et al., 2009)

Strain (cont.)	Classification	Location	Date	Source	Sequence Reference [a,b]
K11	other fermentation	Japan	1981	Shochu sake	(Liti, et al., 2009)
NCYC110	other fermentation	West Africa	pre-1914	Ginger beer from *Zingiber officinale*	(Liti, et al., 2009)
NCYC361	other fermentation	Ireland	1952	Beer spoilage strain from wort	(Liti, et al., 2009)
PW5	other fermentation	Aba, Abia state, Nigeria	2002	Raphia palm wine	*S. cerevisiae* Strain Project
UC5	other fermentation	Kurashi, Japan	pre-1974	Sene sake	*S. cerevisiae* Strain Project
Y12	other fermentation	Ivory Coast	pre-1981	Palm wine	(Liti, et al., 2009)
Y9	other fermentation	Java, Indonesia	pre-1962	Ragi (African or finger millet)	(Liti, et al., 2009)
BC187	wine	Napa Valley, California, USA	NA	Barrel fermentation	(Liti, et al., 2009)
L_1374	wine	Cauquenes, Chile	1999	Fermentation from must Pais	(Liti, et al., 2009)
L_1528	wine	Cauquenes, Chile	1999	Fermentation from must Cabernet	(Liti, et al., 2009)
RM11-1a	wine	California, USA	NA	Vineyard	*S. cerevisiae* RM11-1a Sequencing Project
T73	wine	Alicante, Spain	1987	Monastrel grape in fermentation stage	*S. cerevisiae* Strain Project
WE372	wine	Cape Town, South Africa	NA	Wine	*S. cerevisiae* Strain Project
YIIc17_E5	wine	Sauternes, France	NA	Wine	(Liti, et al., 2009)

[a] *S. cerevisiae* Strain Project. The Genome Institute at Washington University (http://genome.wustl.edu)
[b] *S. cerevisiae* RM11-1a Sequencing Project. Broad Institute of Harvard and MIT (http://www.broad.mit.edu)

Table S7. Putative novel ORFs detected in CRB.

Putative Gene [a]	Length (nt)	Potential Function	% aa Identity	E-val	Species with Best BLAST Hit	Accession
COS2	276	DUP conserved domain, similarity to Scer YBR302C	100%	5.00E-48	*S. cerevisiae JAY291*	EEU05344.1
SNO2/3	669	Glutamine amidotransferase	91%	2.00E-115	*S. cerevisiae* EC1118	CAY80874.1
PDX1	861	Pyridoxine biosynthesis protein	97%	4.00E-144	*S. cerevisiae* RM11-1a, AWRI1631, EC1118, AWRI796, Vin13	EDV12560.1
alpha-Galactosidase	1416	Alpha-galactosidase	98%	0	*S. paradoxus*	CAA64759.1
Me-transferase	858	Methyltransferase	99%	5.00E-156	*S. cerevisiae* JAY291	EEU05580.1
SSF1	1833	Sodium:solute symporter family	99%	0	*S. cerevisiae* JAY291	EEU05581.1
Arginase	864	Arginase	99%	2.00E-154	*S. cerevisiae* JAY291	EEU05582.1
FLR1	1644	Fluconazole resistance protein 1	59%	0	*S. cerevisiae* YJM789, RM11-1a, AWRI1631, JAY291, AWRI796, VL3	EDN64624.1
GAL4-like	1932	GAL4-like transcription factor	99%	0	*S. cerevisiae* JAY291	EEU05583.1
TNA1	1512	High-affinity nicotinic acid plasma membrane permease	100%	0	*S. cerevisiae* EC1118, AWRI796, Lalvin QA3, JAY291	CAY82272.1
5-oxoprolinase	3864	5-oxoprolinase	100%	0	*S. cerevisiae* EC1118	FN295481.1
PUT3	1500	Transcription factor involved in proline utilization	99%	0	*S. cerevisiae* JAY291	EEU07466.1
FLO11	255	Contains a conserved domain from the cell surface flocculin family	100%	2.00E-119	*S. cerevisiae* RM11-1a	EDV12484.1
BIO1	1041	Putative pimeloyl-CoA synthetase; first step of the biotin biosynthesis pathway	98%	0	*S. cerevisiae* A364a	ABQ63082.1
BIO3/6	1314	Putative DAPA synthetase; second or third step of the biotin biosynthesis pathway	99%	0	*S. cerevisiae* Sake yeast kyokai No.7	BAD90000.1
ADH6	1092	NADP-dependent alcohol dehydrogenase	47%	7.00E-93	*Candida glabrata*	XP_447094.1

Putative Gene [a] (continued)	Length (nt)	Potential Function	% aa Identity	E-val	Species with Best BLAST Hit	Accession
LAC1	1611	Lactose permease	99%	0	*S. cerevisiae* JAY291	EEU05584.1
GAL4-like	1671	GAL4-like transcription factor	100%	0	*S. cerevisiae* JAY291	EEU04263.1
NA	396	Conserved hypothetical protein	98%	2.00E-61	*S. cerevisiae* Vin13	EGA76218.1
MPR1	690	N-Acyltransferase superfamily	100%	6.00E-133	*S. cerevisiae* RM11-1a, AWRI1631, EC1118, JAY201, VL3	EDV12565.1
KHR1	939	Heat-sensitive killer toxin	93%	6.00E-156	*S. cerevisiae* FostersO	EGA61940.1
RTM1	930	Molasses resistance protein; confers some resistance to a particular toxic element present in some molasses	99%	6.00E-164	*S. cerevisiae* FL100	P40113.1
STR3	1911	Siderophore-iron transporter	99%	0	*S. cerevisiae* JAY291	EEU04262.1
RSC30	1065	Chromatin structure remodeling complex protein	99%	9.00E-177	*S. cerevisiae*	NP_011923.2
MAL13	1392	Maltose-activator protein; transcriptional activator of MAL gene complex	84%	0	*S. cerevisiae* YJM789	EDN64911.1

[a] Genes that are found in contiguous regions are shaded.

Table S8. Putative novel ORFs detected in LEP.

Putative Gene [a]	Length (nt)	Potential Function	% aa Identity	E-val	Species with Best BLAST Hit	Accession #
AWA1	769	Cell wall protein	72%	2.00E-109	*S. cerevisiae*	AB110100.1
BIO6	240	Biotin biosynthesis enzyme	76%	2.00E-39	*S. cerevisiae* Kyokai No.7	AB200246.1
HPF1	1876	Haze-protective mannoprotein that reduces the particle size of aggregated proteins in white wines	99.8%	0	*S. cerevisiae* EC1118	FN393087.1
MPR1	967	Putative N-acetyltransferase	99.7%	0	*S. cerevisiae* EC1118	FN393087.1
PUT3	1158	Putative transcriptional activator of proline utilization genes	96%	0	*Zygosaccharomyces bailii CLIB213*	FN295481.1
5-oxoprolinase	3864	5-oxoprolinase	99.9%	0	*Zygosaccharomyces bailii CLIB213*	FN295481.1
TNA1	1512	Putative high affinity nicotinic acid plasma membrane permease	99.9%	0	*Zygosaccharomyces bailii CLIB213*	FN295481.1
FLO11	1182	Contains a conserved domain from the cell surface flocculin family	99.9%	0	*Zygosaccharomyces bailii CLIB213*	FN295481.1
COS4	1178	Member of the DUP380 subfamily of conserved, often subtelomerically-encoded proteins	99.7%	0	*Zygosaccharomyces bailii CLIB213*	FN295481.1
SNZ3	393	Putative pyridoxine biosynthesis protein	88%	9.00E-138	*S. cerevisiae S288c*	NM_001179908.1
hypothetical protein	858	unknown	68%	4.00E-07	*Lachancea thermotolerans* CBS 6340	CU928166.1
hypothetical protein	387	Putative glucan alpha-1,4-glucosidase	73%	4.00E-41	*S. cerevisiae S288c*	NM_001179517.1
NUP170	255	Subunit of the inner ring of the nuclear pore complex	92%	2.00E-86	*S. cerevisiae S288c*	NM_001178319.1
MAL2	240	MAL activator for maltose fermentation	100%	4.00E-107	*S. cerevisiae* EC1118	FN393070.1
CFG1	543	Foam promoting protein	70%	8.00E-64	*S. carlsbergensis* 34/70	EU414029.1

[a] Genes that are found in contiguous regions are shaded.

Table S9. Putative novel ORFs detected in MUSH.

Putative Gene [a]	Length (nt)	Potential Function	% aa Identity	E-val	Species with Best BLAST Hit	Accession #
GAL4-like	1221	GAL4-like Zn2Cys6 binuclear cluster DNA-binding domain	97%	0	*S. bayanus* NBRC1948	FR851878.1
RTM1	2519	Molasses resistance protein; confers some resistance to a particular toxic element present in some molasses	99%	3.00E-143	*S. cerevisiae* FL100	U02618.1
MPR1	1372	N-Acyltransferase superfamilty	98%	0	*S. cerevisiae* Sigma1278b	AB031349.1
MPR3	690	Azetidine-2-carboxylic acid acetyltransferase	100%	0	*S. cerevisiae* EC1118	FN393087.1
SUC1	329	Invertase; sucrose hydrolyzing enzyme	97%	4.00E-122	*S. cerevisiae*	X07570.1
SUC4	1165	Invertase; sucrose hydrolyzing enzyme	99%	4.00E-141	*S. cerevisiae*	X07572.1
HPF1	301	Haze-protective mannoprotein; reduces the particle size of aggregated proteins in white wines	99%	5.00E-146	*S. cerevisiae* EC1118	FN393087.1
MAL33	357	MAL activator for maltose fermentation	100%	4.00E-180	*S. bayanus* NBRC2031	FR820653.1
MAL13	279	MAL activator for maltose fermentation	99%	7.00E-137	*S. bayanus* NBRC1948	FR754539.1
DUP240	708	Putative integral membrane protein	74%	2.00E-116	*S. cerevisiae* CLIB413	AJ586498.1
DUP240	723	Putative integral membrane protein	80%	2.00E-180	*S. cerevisiae* YIIc17	AJ586495.1
SCC2	132	Subunit of cohesin loading factor (Scc2p-Scc4p); a complex required for loading of cohesin complexes onto chromosomes	88%	3.00E-16	*S. cerevisiae* S288c	NM_001180488.1

[a] Genes that are found in contiguous regions are shaded.

Table S10. Transcripts from DBY, MUSH, LEP and CRB not aligned to S288c transcripts.

Strain	Number of Transcripts	Not Present in S288c Genome
DBY	103	56 (54%)
MUSH	114	29 (25%)
LEP	129	40 (31%)
CRB	148	65 (44%)

Table S11. Genes with strain-specific expression effects in DBY, MUSH, LEP and CRB that overlap with genes required for ethanol tolerance from previous studies (Gibney, et al., 2013; Kubota, et al., 2004; Teixeira, et al., 2009; van Voorst, et al., 2006; Yoshikawa, et al., 2009).

Gene	Name	Function
**DBY**		
YAL044C	*GCV3*	H subunit of the mitochondrial glycine decarboxylase complex
YBL072C	*RPS8A*	Protein component of the small (40S) ribosomal subunit
YBR026C	*ETR1*	2-enoyl thioester reductase
YBR126C	*TPS1*	Synthase subunit of trehalose-6-P synthase/phosphatase complex
YBR291C	*CTP1*	Mitochondrial inner membrane citrate transporter
YCL036W	*GFD2*	Protein of unknown function
YDR074W	*TPS2*	Phosphatase subunit of the trehalose-6-P synthase/phosphatase complex
YDR127W	*ARO1*	Pentafunctional arom protein
YDR173C	*ARG82*	Inositol polyphosphate multikinase (IPMK)
YDR174W	*HMO1*	Chromatin associated high mobility group (HMG) family member
YDR529C	*QCR7*	Subunit 7 of the ubiquinol cytochrome-c reductase complex
YER090W	*TRP2*	Anthranilate synthase
YER154W	*OXA1*	Mitochondrial inner membrane insertase
YGL035C	*MIG1*	Transcription factor involved in glucose repression
YGL237C	*HAP2*	Subunit of the Hap2p/3p/4p/5p CCAAT-binding complex
YGR102C	*GTF1*	Subunit of the trimeric GatFAB AmidoTransferase(AdT) complex
YHR065C	*RRP3*	Protein involved in rRNA processing
YIL026C	*IRR1*	Subunit of the cohesin complex
YIL124W	*AYR1*	NADPH-dependent 1-acyl dihydroxyacetone phosphate reductase
YJL177W	*RPL17B*	Ribosomal 60S subunit protein L17B
YKL068W	*NUP100*	FG-nucleoporin component of central core of the nuclear pore complex
YKL109W	*HAP4*	Subunit of the Hap2p/3p/4p/5p CCAAT-binding complex
YKL113C	*RAD27*	5' to 3' exonuclease, 5' flap endonuclease
YLR027C	*AAT2*	Cytosolic aspartate aminotransferase
YLR260W	*LCB5*	Minor sphingoid long-chain base kinase
YLR270W	*DCS1*	Non-essential hydrolase involved in mRNA decapping
YLR304C	*ACO1*	Aconitase
YLR372W	*SUR4*	Elongase
YLR414C	*PUN1*	Plasma membrane protein with a role in cell wall integrity
YML034W	*SRC1*	Inner nuclear membrane protein
YMR015C	*ERG5*	C-22 sterol desaturase
YMR032W	*HOF1*	Bud neck-localized, SH3 domain-containing protein required for cytokinesis
YMR145C	*NDE1*	Mitochondrial external NADH dehydrogenase

**DBY (continued)**		
YMR179W	*SPT21*	Protein with a role in transcriptional silencing
YNL098C	*RAS2*	GTP-binding protein
YNL220W	*ADE12*	Adenylosuccinate synthase
YNR041C	*COQ2*	Para hydroxybenzoate: polyprenyl transferase
YOL076W	*MDM20*	Non-catalytic subunit of the NatB N-terminal acetyltransferase
YOR196C	*LIP5*	Protein involved in biosynthesis of the coenzyme lipoic acid
YOR375C	*GDH1*	NADP(+)-dependent glutamate dehydrogenase
YPL157W	*TGS1*	Trimethyl guanosine synthase, conserved nucleolar methyl transferase
YPL212C	*PUS1*	tRNA:pseudouridine synthase
YPR160W	*GPH1*	Glycogen phosphorylase required for the mobilization of glycogen
**MUSH**		
YAL037W		Putative protein of unknown function
YBR072W	*HSP26*	Small heat shock protein (sHSP) with chaperone activity
YBR101C	*FES1*	Hsp70 (Ssa1p) nucleotide exchange factor
YCL029C	*BIK1*	Microtubule-associated protein
YCL036W	*GFD2*	Protein of unknown function
YDL066W	*IDP1*	Mitochondrial NADP-specific isocitrate dehydrogenase
YDR300C	*PRO1*	Gamma-glutamyl kinase
YEL044W	*IES6*	Protein that associates with the INO80 chromatin remodeling complex
YEL046C	*GLY1*	Threonine aldolase
YER090W	*TRP2*	Anthranilate synthase
YGL148W	*ARO2*	Bifunctional chorismate synthase and flavin reductase
YGR260W	*TNA1*	High affinity nicotinic acid plasma membrane permease
YHR065C	*RRP3*	Protein involved in rRNA processing
YIL053W	*RHR2*	Constitutively expressed glycerol-1-phosphatase
YKL110C	*KTI12*	Protein that plays a role in modification of tRNA wobble nucleosides
YKL211C	*TRP3*	Indole-3-glycerol-phosphate synthase
YKR093W	*PTR2*	Integral membrane peptide transporter
YLR056W	*ERG3*	C-5 sterol desaturase
YNL064C	*YDJ1*	Type I HSP40 co-chaperone
YPL061W	*ALD6*	Cytosolic aldehyde dehydrogenase
**LEP**		
YBR072W	*HSP26*	Small heat shock protein (sHSP) with chaperone activity
YBR291C	*CTP1*	Mitochondrial inner membrane citrate transporter
YHR065C	*RRP3*	Protein involved in rRNA processing
YLR386W	*VAC14*	Enzyme regulator
YNL220W	*ADE12*	Adenylosuccinate synthase
**CRB**		
YCL036W	*GFD2*	Protein of unknown function
YDR035W	*ARO3*	3-deoxy-D-arabino-heptulosonate-7-phosphate (DAHP) synthase
YDR127W	*ARO1*	Pentafunctional arom protein
YER090W	*TRP2*	Anthranilate synthase
YHR065C	*RRP3*	Protein involved in rRNA processing
YNL220W	*ADE12*	Adenylosuccinate synthase
YPL061W	*ALD6*	Cytosolic aldehyde dehydrogenase

Table S12. Short read sequencing statistics.

Strain	Libraries	Total Reads	Filtered Reads				Callable Bases	Cov.
			Bad Mate	Duplicate	Unmapped	Total Filtered		
CRB	2	65,849,073	602,583	7,170,294	521,532	8,294,409	12,142,086	360x
LEP	2	99,133,756	748,599	14,013,893	1,001,319	15,763,811	12,145,247	522x
MUSH	1	46,947,752	453,898	4,521,843	633,227	5,608,968	12,141,639	259x

Table S13. *De novo* assembly statistics.

Strain	k	m	Contigs	Scaffolds	Singleton Scaffolds	Total Bases (Mbp)	Max Scaffold (bp)	N50 Scaffold (bp)	Mean Scaffold (bp)
CRB	45	55	3,174	795	322	11.25	184,004	47,643	14,176
LEP	45	65	1,164	371	123	11.42	304,277	76,733	30,786
MUSH	41	55	4,478	1,108	495	11.19	170,565	31,318	10,126
